# Reconstitution reveals how myosin-VI self-organises to generate a dynamic mechanism of membrane sculpting

**DOI:** 10.1038/s41467-019-11268-9

**Published:** 2019-07-24

**Authors:** Benoit Rogez, Laeschkir Würthner, Anastasiia B. Petrova, Felix B. Zierhut, Dario Saczko-Brack, Maria-Ana Huergo, Christopher Batters, Erwin Frey, Claudia Veigel

**Affiliations:** 1 Department of Cellular Physiology, Biomedical Center (BMC), Ludwig-Maximilians-Universität München, Grosshadernerstrasse 9, 82152 Planegg-Martinsried, Germany; 2 Center for Nanosciences (CeNS), Ludwig-Maximilians-Universität München, Schellingstrasse 4, 80799 München, Germany; 3 Arnold-Sommerfeld-Center for Theoretical Physics (ASC), Department of Physics, Ludwig-Maximilians-Universität München, Theresienstrasse 37, 80333 München, Germany; 4 Theoretical and Applied Physical Chemistry Research Institute (INIFTA), National University of La Plata, Diagonal 113, 1900 La Plata, Argentina

**Keywords:** Membrane biophysics, Motor proteins

## Abstract

One enigma in biology is the generation, sensing and maintenance of membrane curvature. Curvature-mediating proteins have been shown to induce specific membrane shapes by direct insertion and nanoscopic scaffolding, while the cytoskeletal motors exert forces indirectly through microtubule and actin networks. It remains unclear, whether the manifold direct motorprotein–lipid interactions themselves constitute another fundamental route to remodel the membrane shape. Here we show, combining super-resolution-fluorescence microscopy and membrane-reshaping nanoparticles, that curvature-dependent lipid interactions of myosin-VI on its own, remarkably remodel the membrane geometry into dynamic spatial patterns on the nano- to micrometer scale. We propose a quantitative theoretical model that explains this dynamic membrane sculpting mechanism. The emerging route of motorprotein–lipid interactions reshaping membrane morphology by a mechanism of feedback and instability opens up hitherto unexplored avenues of membrane remodelling and links cytoskeletal motors to early events in the sequence of membrane sculpting in eukaryotic cell biology.

## Abstract (web summary)

Curvature-mediating proteins are known to induce specific membrane shapes, but whether motorprotein-lipid interactions remodel membranes too remains unclear. Here authors show that curvature-dependent lipid interactions of myosin-VI remodel the membrane geometry into dynamic spatial patterns.

## Introduction

In eukaryotes, the morphology of cells, organelles and membrane domains is critically dependent on the membrane curvature. Active remodelling of the membrane curvature is the key to cellular motile processes, including endocytosis, cell migration and polarisation in morphogenesis and development^1,2^. Curvature-mediating proteins have been shown to induce specific membrane shapes by direct insertion and nanoscopic scaffolding^3–5^. Since fundamental concepts and the biological relevance of membrane curvature were first recognised over 40 years ago^6,7^, it was understood that the cytoskeleton plays a more indirect role by generating a macroscopic scaffold to exert forces onto the plasma membrane and intracellular membrane systems via actin and microtubule polymerisation and motor protein activity^8^. It remained unexplored, however, whether the manifold direct interactions between lipid-binding motor proteins and the target membranes themselves affect the membrane curvature^9–11^, and if so, how? The lipid-binding molecular motor myosin-VI (myo6) functions in a wide range of cellular processes that involve not only dramatic changes in local membrane shape, including endocytosis, polarized secretion, Golgi re-organisation and autophagy^9,12–14^, but also cell migration and invasion in numerous cancers^15–17^. Myo6, the only myosin class that has been shown to move towards the minus end of actin filaments, has been reported to bind directly to lipid membranes via its C-terminal tail domain^9^. The molecular mechanisms of this protein–lipid interaction remained unclear^9,18^.

Using reconstituted, fluid supported lipid bilayers, super-resolution fluorescence microscopy and membrane-reshaping gold nano-particles, we show that myo6 on its own remarkably remodels the membrane to form rugged, flower-shaped membrane pores. We find that the curvature-dependent and cooperative binding kinetics of the myosin favour saddle-shaped membrane geometries, which leads to characteristic and growing spatial patterns. We propose a quantitative theoretical model that describes this innovative route of protein–membrane interaction and ensuing membrane morphology, which we call a dynamic membrane sculpting mechanism. Our findings highlight a previously unnoticed basic feature of protein–lipid interactions that opens up unexplored avenues for the shaping of the plasma membrane and intracellular organelle systems in eukaryotes.

## Results

### Myosin-VI reshapes the protein–lipid interface

**Fig. 1:**
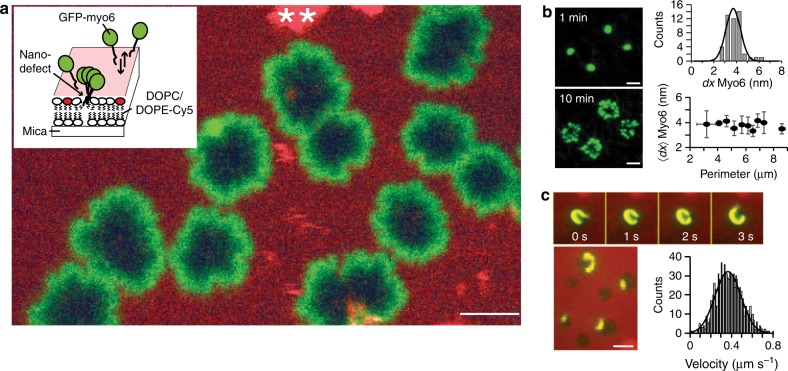
Myo6 reshapes transient membrane defects into growing pores. **a** Schematic of the in vitro assay with 150 nM green fluorescent protein (GFP)-myo6 (green) in solution interacting with a 1,2-dioleoyl-sn-glycero-3-phosphocholine (DOPC):1,2-dioleoyl-sn-glycero-phosphoethanolamine-*N*-Cyanine 5 (DOPE-Cy5) (red) fluid lipid bilayer (4000:1 ratio) imaged using confocal microscopy; image after 30 min; scale bar 5 µm. **b** Growth of flower-shaped membrane pores 1 and 10 min after addition of 150 nM myo6, with a GFP-myo6 (green):myo6 ratio of 1:10 and the DOPC bilayer unlabelled; scale bars 2 µm. Average distance between myo6 molecules lining the flower *〈dx〉* ~ 3.74 ± 0.64 nm (mean ± s.d., *R*^2^ = 0.92, *n* = 48 flowers, perimeters 2–9 µm), independent of the perimeter of the flower (total internal reflection fluorescence microscopy). Same data plotted as a single distribution and sorted by perimeter (mean ± s.d.). **c** Myo6 motors (unlabelled) bound to a DOPC:DOPE-Cy5 (red) fluid lipid bilayer (4000:1 ratio) translocate Alexa 488-labelled actin filaments (yellow) at an average velocity 365 ± 125 nm s^−1^ (mean ± s.d., *n* = 153 filaments) along the perimeter of the flower (2 mM ATP); scale bar 3 µm. Source data are provided as a Source Data file

We investigated the effect of fluorescently labelled myo6 in solution on the shape of a reconstituted fluid model membrane consisting of a phosphatidylcholine lipid bilayer, a major constituent of the plasma and organelle membranes in animals and plants^19,20^, and a well-characterised model system^21^ (Fig. 1a). The lipid diffusion coefficient *D* ~ 1.15−1.8 μm^2^ s^−1^, determined by fluorescence recovery after photobleaching (FRAP) in the absence and presence of myo6 (Supplementary Fig. 1a, b), confirmed the fluidity of the solid supported and intrinsically flat 1,2-dioleoyl-sn-glycero-3-phosphocholine (DOPC) bilayer^22,23^. Unexpectedly, we found that the myosin-binding events to the bilayer were very sparse but highly cooperative, consistent with the motor molecules binding to a small number of spontaneously and transiently forming nanometre-sized membrane defects or transversal membrane pores^24,25^. The binding process caused the pores to grow into rugged flower shapes, reaching several microns in diameter after about 30 min (at 150 nM myo6 in solution). The lipids displaced from the perimeter of the pores to accommodate myosin binding were redeposited onto the membrane as micelles or small vesicles (double asterisks (**) in Fig. 1a), revealing a heretofore unknown process of motor protein–lipid interaction. Using a quantitative analysis of the total internal reflection fluorescence microscopy (TIRFM) data (Fig. 1b and Supplementary Fig. 1c, d) and a 10:1 ratio of myo6:green fluorescent protein (GFP)-myo6 to ensure the fluorescence signal measured for each flower was within the linear range of the EMCCD camera, we estimated the density of the myo6 motors at the protein–lipid interface. By measuring the perimeter of the flowers, their total fluorescence intensity and the fluorescence signal of single GFP-myo6 molecules in TIRF mode, we estimated an average distance of 〈*dx*〉 ~ 3.74 ± 0.64 nm (mean ± s.d.) between neighbouring myosin molecules along the perimeter of the growing flowers (see ‘Methods’). The value *dx* was independent of the actual perimeter of the pore and indicated a dense packing, given the physical size of the myosin molecules (diameter ~ 3–5 nm^18,26^). The myo6 molecules might well interact with each other and arrange themselves in a more complex geometry than in a monolayer. We then investigated the mechanical properties of these ensembles of densely packed myosin and found that they were mechanically fully functional. They translocated Alexa 488-labelled actin filaments at 365 ± 125 nm s^−1^ (mean ± s.d., *n* = 153 filaments) at saturating ATP concentrations (Fig. 1c, actin in yellow, 2 mM ATP, 22 ^o^C), at least as fast as myo6 monomers and dimeric constructs in vitro and in cells^27–29^. The propensity to self-organise to generate membrane pores was also observed on giant unilamellar DOPC vesicles (Supplementary Fig. 1e), which confirmed that this cooperative membrane interaction of myo6 was not restricted to the flat supported bilayer but an intrinsic property of the motor–lipid interaction.

### Myosin-VI binding to a lipid bilayer is curvature sensitive

**Fig. 2:**
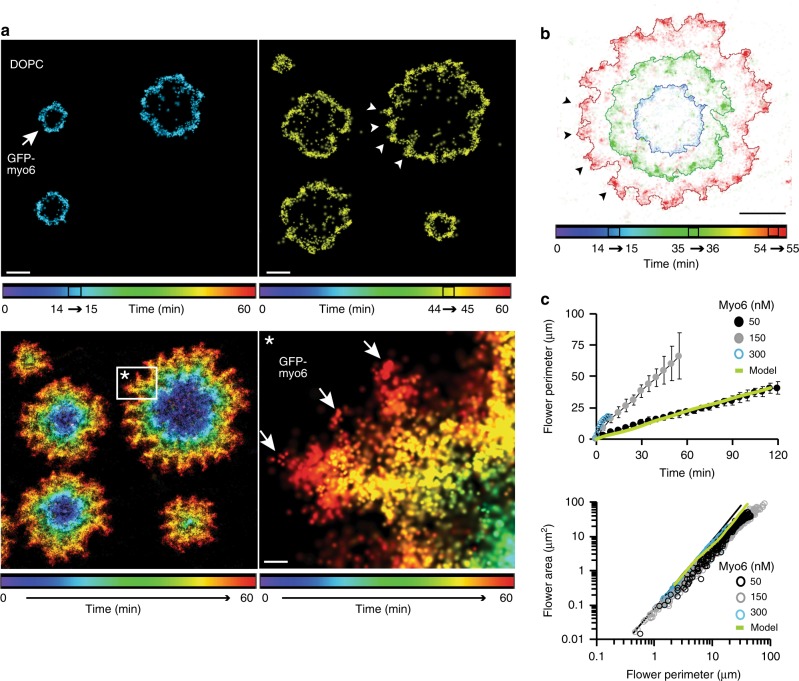
Myo6 binding to a lipid bilayer is curvature sensitive. **a** Super-resolution microscopy (SRM) of myo6 flowers growing over 60 min (150 nM myo6, 10% green fluorescent protein (GFP)-myo6; bilayer unlabelled). The colour-coded time stamps were obtained from SRM images integrated over 1 min time periods (14–15 and 44-45 min, respectively), bilayer unlabelled. Arrow heads mark hotspots of GFP-myo6 binding. The SRM image integrated over 60 min shows the new appearance and growth of the myo6 flowers with the colour encoding the progress in time. Zoomed-in image shows freshly bound myo6 (red) at hotspots (arrows) where the membrane border bulges outward, away from the pore centre; scale bars 1 µm; scale bar in inset (*) 200 nm. **b** GFP-myo6 detected during three colour-coded, 1 min time intervals superimposed onto the respective perimeter of the flowers (solid lines, ‘Methods’). Hotspots of GFP-myo6 marked by arrow heads; scale bar 1000 nm. **c** Perimeter determined by integrating SRM data over time *t*; *n* = 253, 561 and 948 measurements in total for 50 (black filled circles), 150 (grey filled circles) and 300 nM myo6 (blue unfilled circles) from 20 to 70 flowers in each condition. Same data shown as scatter plot (area vs perimeter) and as mean perimeter (mean ± s.d.) sorted by time *t*. Green line, theoretical model (see below). Source data are provided as a Source Data file

To uncover the mechanisms underlying pore growth and the emergent flower-like morphology of the motor–lipid interface, we applied super-resolution microscopy (SRM) and localised single GFP-myo6 molecules at ~ 25nm spatial resolution (‘Methods’). The colour-coded time stamps in Fig. 2a, b obtained from SRM images integrated over 1 min time periods showed the new appearance and growth of myo6 flowers during a 60-min time interval (150 nM myo6 in solution, 10% GFP-myo6). The perimeter of the flowers grew at a roughly constant rate, directly proportional to the myo6 concentration in solution (Fig. 2c). Combined with the constant density of myo6 along the growing perimeter (Fig. 1b), this result indicated a mechanism with an—on average—constant rate of myosin binding to and lipid displacement from the protein–lipid interface. Strikingly, the SRM images also exposed local hotspots of myo6 attachment (arrow heads), which formed a quite regular spatial pattern along the growing perimeter of the flowers in the time stamp overlay. Inspection of the hotspots in the zoomed-in image indicated that the freshly bound GFP-myo6 molecules (red, arrows) were incorporated preferentially at regions where the membrane was bulging outward, away from the centre of the flower; there the membrane geometry is saddle-shaped (negative Gaussian curvature). Superposition of all GFP-myo6 molecules, detected during different time stamps, over the respective perimeter of the flower (Fig. 2b) demonstrated that the hotspots indeed co-localised with the protrusions of the perimeter (arrows heads). This showed that the process of myosin binding was cooperative and strongly sensitive to the membrane curvature. The curvature dependence of the myosin binding was also reflected in the roughness of the perimeter, so that the ratio of flower area vs perimeter deviated slightly from a perfect circle growing over time (Fig. 2c, black curve).

### Myosin-VI favours a saddle-shaped membrane geometry

**Fig. 3:**
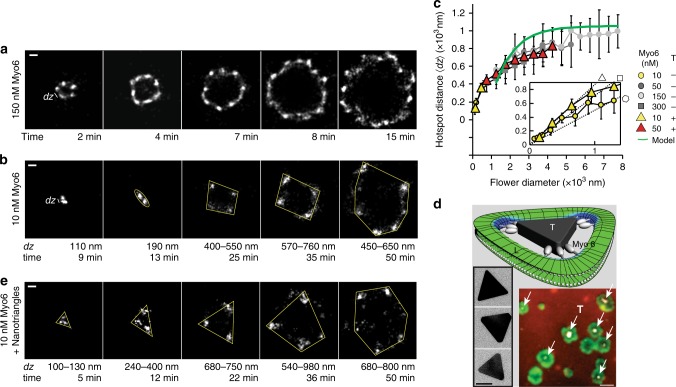
Myo6 lipid binding favours a saddle-shaped membrane geometry. Representative super-resolution microscopic (SRM) images at **a** 150 nM and **b** 10 nM myo6, integrated over 1 min; scale bar **a** 400 nm and **b** 200 nm. Distance *dz* marks distances between GFP-myo6 hotspots. **c** Distances *〈dz〉* (mean ± s.d.) from 10 to 36 measurements in the absence or presence of nano-triangles T (0.3 pM), respectively. Green line, theoretical model. Inset: dotted lines show the side lengths of an equilateral triangular, quadrangular and hexagonal shape with increasing diameter of 0–1 µm. **d** Transmission electron microscopic images of gold nano-triangles T (side length 60–80 nm, height 8 nm), scale bar 40 nm; cartoon of a lipid bilayer surrounding a T (L = lipid, green; saddle-shaped curvature, blue); cartoon illustrates that, in contrast to a circular-shaped membrane pore, triangular-shaped pores have saddle-shaped curvatures only at the corners of the triangular pore (blue). This is where we found the curvature-sensitive myo6 to bind (see **e**); gap between lipid and nano-triangle not to scale and enlarged for clarity. Co-localisation of the TRITC-labelled T (white, arrows) and GFP-myo6 (green) on a DOPC:DOPE-Cy5 bilayer (red), scale bar 2 µm. **e** SRM of a flower growing at 10 nM myo6 in the presence of 0.3 pM T; scale bar 200 nm. Source data are provided as a Source Data file

Next, we set out to characterise the morphology of the motor–lipid interface by SRM (Fig. 3a) using an intensity threshold to separate the hotspots of myosin binding from the surrounding fluorescence signal (Supplementary Fig. 2). The distances *dz* between the hotspots were determined for different myo6 concentrations in solution. At 10 nM myo6, the speed of flower growth was slow enough to resolve the early stages of hotspot formation (Fig. 3b). As for all different myo6 concentrations and speeds of flower growth, the hotspots were almost equally spaced with an average distance *〈dz〉* saturating at ~900 nm (Fig. 3c), we concluded that this regular myosin pattern is likely an intrinsic feature of the process underlying membrane reshaping by myo6 in our two-component model system (myo6, DOPC). In particular, these observations hinted at a feedback between myosin binding and membrane curvature.

To address (and quantify) this possible feedback, we studied how the shape of the initial nano-pore affected the myo6 binding. To this end, we used gold nano-triangles^30^ with a side length of 60–80 nm and a height of ~8 nm (Fig. 3d, transmission electron microscopy (TEM)), which were deposited on the mica surface before the bilayer was applied. With their sufficiently small aspect ratio, the nano-triangles were not covered by the bilayer surrounding them^31^. FRAP studies confirmed bilayer fluidity in their presence (Supplementary Fig. 3). The nano-triangles acted as seeds for myosin insertion into the bilayer, as shown in the three-colour confocal fluorescence experiment (Fig. 3d nano-triangles T, white arrows). During the first 5 min following addition of 10 nM myo6, the triangular binding pattern intriguingly indicated that myo6 inserted nearly exclusively near the tips of the gold nano-triangles (Fig. 3e). The smallest distances of ~100 nm between the earliest myo6 hotspots (Fig. 3e) forming a triangular shape indicated that initially the proximity between bilayer and nano-triangle (60–80 nm side length) must have been ≤20 nm. Over ~20 min, the triangular shape and orientation of the pattern was preserved, as the flower diameter expanded (Fig. 3c inset), until additional myo6 hotspots emerged and the ‘memory’ of the initial pore shape was lost. The experiments confirmed that myo6 strongly favoured the saddle-shaped membrane geometry (negative Gaussian curvature)^5^ at the tips of the triangular pore over the sides of the pore (cartoon). Importantly, the final characteristic distance between the hotspots was similar to the one observed in the absence of the triangles (Fig. 3c).

### A quantitative model explains the dynamic membrane sculpting

**Fig. 4:**
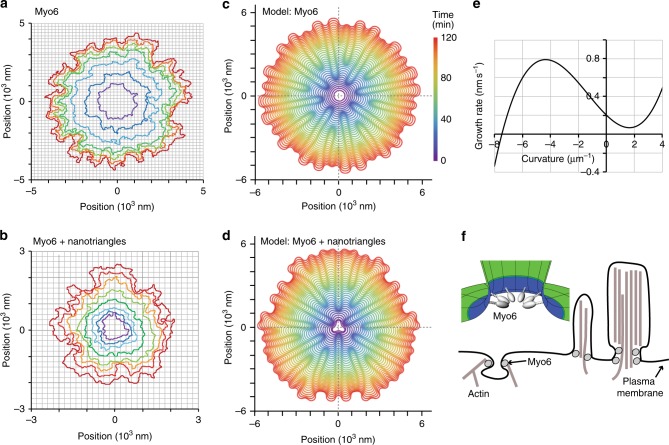
A quantitative model explains the dynamic membrane sculpting. **a** Representative examples of the flower perimeter determined by super-resolution microscopy at different time stamps in the absence and **b** presence of a nano-triangle inserted into the membrane as a seed (50 nM myo6, colour code as in Fig. 2). **c** The model reproduces the time evolution of the flower perimeter for different starting geometries in the absence and **d** presence of nano-triangles. **e** Profile of the curvature-dependent myo6-binding rate used in the model. **f** Localisation of myo6 at cellular sites with saddle-shaped membrane geometry^13^

We integrated the above experimental observation to propose a quantitative theoretical model for this emerging form of dynamic membrane sculpting. The model describes the dynamics of the experimentally determined protein–lipid interface (Fig. 4a, b) as the time evolution of a closed (planar) curve *Γ*(*t*) (Fig. 4c, d), which we represent by the position vector ${\vec{\mathbf{x}}}\left( {t,\sigma } \right) = \left( {x\left( {t,\sigma } \right),y\left( {t,\sigma } \right)} \right)$ (‘Methods’, Supplementary Fig. 4), where *t* denotes time and *σ* parametrises the position along the curve *Γ*(*t*). Our basic assumption is that the dynamics of the interface is determined by the normal velocity *V*_n_,

1 $$\begin{array}{*{20}{c}} {\partial _t{\vec{\mathrm{x}}} \cdot {\hat{\mathrm{n}}} = V_{\mathrm{n}},} \end{array}$$

where ${\hat{\mathrm{n}}}$ is the outward unit normal vector on *Γ*(*t*) (‘Methods’, Supplementary Fig. 4). The growth velocity *V*_n_ is determined by the interface morphology, i.e. the local interface curvature *κ* and its spatial modulation: $V_{\mathrm{n}} = f\left( \kappa \right) - \alpha \nabla ^2\kappa$ (see ‘Methods’ section for a detailed derivation). The observed feedback between interface curvature and myosin binding is accounted for by a curvature-dependent growth rate *f (κ*), shown in Fig. 4e, which we obtained by (systematically) fitting the measured time dependence of the average flower radius to our theoretical description (Fig. 2c, ‘Methods’, Supplementary Fig. 6). As in the experiments, the interface growth speed is enhanced at negative and reduced at positive curvatures with respect to the average value. This theoretical approach for the dynamics of the protein–lipid interface is different from force balance arguments used to describe the dynamics of pore nucleation and growth in giant unilamellar vesicles (GUVs) or lipid membranes^32–34^. The main difference arises from the positive feedback of myo6 binding to regions of negative curvature (recruitment), which is the major driving force of the dynamics. Dynamics based on this feedback alone would lead to rough protein–lipid interfaces with no emerging characteristic length scale. This roughening tendency is counteracted by molecular processes including myosin rearrangement on the membrane and line tension of the lipid bilayer that smoothen the interfaces similar to models for crystal growth^35–38^; for a more detailed discussion and an illustration, see ‘Methods’ section and Supplementary Fig. 5a, b. We effectively account for these processes by a term, *α*∇^2^*κ*, which is acting as an effective line tension term penalising changes in the interface curvature. This can be seen by rewriting the growth law in ‘potential form’,

2 $$\begin{array}{*{20}{c}} {V_{\mathrm{n}} = \mu \frac{{\delta {\cal{F}}}}{{\delta \kappa }},} \end{array}$$

where *μ* denotes an interface mobility. The effective free energy functional is given by

3 $$\begin{array}{*{20}{c}} {F = \mathop {\int}\nolimits_\Gamma {\left[ {F\left( \kappa \right) + \frac{\gamma }{2}\left( {\partial _s\kappa } \right)^2} \right]{\mathrm{d}}s} } \end{array}$$

with the second term penalising gradients in curvature. The corresponding stiffness parameter *γ* is related to the above phenomenological parameter *α* through the mobility, *α* = *μ*·*γ*. The function $F\left( \kappa \right) \propto {\int} {{\mathrm{d}}\kappa f\left( \kappa \right)}$ may be interpreted as an effective curvature potential. Since we model line tension only effectively via the term $- \alpha \partial _s^2\kappa$, the parameter *α* is proportional but not equal to a classical line tension parameter^35–37^. This is also evident from the units of the parameter *α* (length^4^ time^−1^), which is different from a classical line tension (energy length^−1^).

After validating the model parameters for the growth term (‘Methods’, Supplementary Fig. 6, Fig. 4e) (see above), we performed an extensive set of simulations in order to explore whether the model can qualitatively as well as quantitatively explain the dynamic morphology of the protein–lipid interface observed experimentally (Fig. 4a, b). In fact, it correctly reproduces the key qualitative features of the experimentally determined protein–lipid interface (Fig. 4a–d, ‘Methods’, Supplementary Fig. 6): flower-shaped morphology, memory effects for triangle-shaped pores, spontaneous emergence of new hotspots, and tip-splitting. To quantiatively match the average distance dz between the experimentally observed hotspots of myo6 attachment (Fig. 3c), we fitted the value of the effective line tension parameter: *α* = 10^−6^ μm^4^ s^−1^ (‘Methods’). Taken together, we conclude that the basic mechanisms included in the model, feedback between binding kinetics of myo6, interface growth and surface smoothing mediated by myo6 rearrangement and line tension of the bilayer, explain the observed dynamics of the membrane sculpting process.

## Discussion

Combining experimental approaches and theoretical modelling, our study addresses a fundamental problem of molecular cell biology, which is to reveal mechanisms used by cells to produce and remodel membrane shape. The sequence of events during membrane reshaping in various cell biological contexts remains controversial. Molecular motors are thought to get involved at the later stages of membrane trafficking or endocytosis, while other, specialised proteins are responsible for initial local membrane reshaping. Our results challenge the classical view of the role of motor proteins and introduce an innovative function of membrane sculpting by direct protein–lipid interaction, indicating that motor proteins can get involved in the initial stages of membrane remodelling.

Using a fluid supported lipid bilayer, we found that the molecular motor myo6 on its own remarkably remodels the membrane into dynamic spatial patterns. The motor protein–lipid interaction generates a heretofore unknown system of feedback and instability, so that dynamic membrane patterns self-organise on the nanometre to micrometre length scale. The process starts with myo6 binding to spontaneously and transiently forming membrane pores. Subsequently, these pores grow into rugged flower shapes, reaching several microns in diameter. We have developed a theory that quantitatively explains the membrane sculpting process leading to the dynamic morphology of the growing protein–lipid interface, including the spontaneous emergence of new myo6-binding hotspots and tip splitting. The basic idea underlying our theory is a reduction of the complex and interlinked chemo-mechanical problem of protein (myo6) binding kinetics and dynamics of the protein–lipid interface to an effective description in terms of an evolving line characterised by its local curvature and gradients in that curvature. The nonlinear coupling between the morphology of the protein–lipid interface and biochemical processes facilitating binding and unbinding of proteins is encoded in a curvature-dependent growth term that is inferred from the experimental data. In addition to being computationally efficient, this theoretical approach provides innovative insights: It shows that local curvature-dependent interactions between myo6 and lipids are sufficient to remodel the membrane, explaining the dynamic membrane sculpting. The theory also reveals that the pattern forming process is mainly controlled by material parameters like the effective line tension and membrane stiffness. This suggests that length scale selection in the membrane pattern depends crucially on the lipid composition and the molecular interactions of myo6 with the lipids, which to date are not fully understood. Moreover, our theoretical approach is quite general and can easily be extended to include nonlocal interactions and coupling to other dynamic variables, such as the myo6 bulk density. Therefore, we expect that the theoretical concepts and methods developed here may be broadly applicable to biological processes dynamically coupling membrane morphology and biochemical pattern formation. This may serve as a basis for future studies addressing general pattern forming processes on evolving geometries in biological systems.

Our results also show that membrane remodelling by myo6 does not require nucleotide as an energy source nor interactions with actin. The tail domain of the motor was sufficient to induce growing membrane pores, indicating that the catalytic activity of the motor domain is not necessary for myo6 to induce the remodelling effect on the membrane shape. Furthermore, the time course of pore growth and the characteristic myo6binding patterns were similar in the absence or presence of apyrase treatment of the motor protein to remove residuals of ATP or ADP (Supplementary Fig. 1f–h). These results are consistent with the binding energy of the myo6–lipid interaction to be sufficient for the remodelling of the membrane.

The densities of the motor protein obtained by self-organisation at the protein–lipid interface was high, with an average distance between myosin motors of only ~3.74 ± 0.64 nm, independent of the actual perimeter of the growing protein–lipid interface. These densities, which appear to be at the limit for spacing if all myosin molecules in the flower are bound to the membrane, might well indicate interaction between the lipid-bound motors, affecting their orientation on the bilayer, so that the mechanical output of the motor protein is optimised when interacting with actin filaments. In fact, the native myo6 motors, bound to the pores of the DOPC lipid bilayer, translocated actin filaments at  ~365 ± 125 nm s^−1^ (22 °C) at saturating ATP concentrations (2 mM ATP). These velocities exceed the range of speeds previously reported for monomeric and enforced dimeric myo6 constructs^18,27,29,39^. The variations in speed might be related to the variability of motors partly back-folded and/or contributing with a range of lever arm lengths to the gliding velocity in these studies. The details of the molecular conformation of myo6 when bound at high density to the lipid bilayer in our study, which produced these high actin-gliding velocities, remain unclear and will be addressed in future experiments.

Our study also revealed the unexpected result that myo6–lipid binding does not require the presence of PIP2 lipids^9^. Instead, the myo6–lipid interaction depended on the shape of the bilayer and was highly selective for saddle-shaped membrane geometries (Fig. 4f). The assay of triangular-shaped gold nano-particles combined with SRM has now provided the opportunity to explore the geometric preference of the (motor-)protein–lipid interaction at the nanometre scale. The strong preference of myo6 for saddle-shaped membrane geometries is consistent with its intracellular locations at the base of microvilli, stereocilia and sites of endocytosis (Fig. 4f)^13^.

In conclusion, our results broaden the classical view of the role of motor proteins and introduce the function of membrane sculpting by direct protein–lipid interaction. In addition to the currently known distinct mechanisms that allow proteins to sense, stabilize or generate high local membrane curvature, we believe that we have identified a hitherto unknown mechanism of dynamic membrane sculpting by (motor-)protein–lipid interaction. Consistent with the generality of the already identified mechanisms of membrane reshaping within eukaryotic cells, our original assay and modelling approach might help to uncover additional mechanisms underlying membrane shaping in the near future and contribute towards revealing the universal role of membrane curvature in cellular functions.

## Methods

### Molecular biology

Myosin-VI (myo6) from human cells (accession number XP_005248783.1, residues 1–1253) was cloned into pFastbacHtB (Invitrogen) via EcoRI/SalI sites using primers HVIEcoRIF (GAATTCAAATGGAGGATGGAAAGCCCG) and HVISalR (GTCGACTTATTTCAACAGGTTCTGC). Two full-length constructs were expressed, one native full-length myo6 and a fusion full-length myo6 with an N-terminal eGFP cloned between the BamHI site using primers GFPBamF (GGATTCATGGTGAGCAAGGGCGAG) and GFPBamR (GAATCCCTTGTACAGCTCGTCCATG); furthermore, a Head construct (aa1–913) was cloned into the pFastbacHTb vector via EcoRI/SalI sites using primers HVIEcoRIF and HVI913SalR (GTCGACTTACATGGACGAGCTGTACAAGGGATTC), note HVIEcoRIF has a stop codon added. A Tail construct was cloned into pET28a vector via EcoRI/SalI (aa1037–1253) using primers HVI1037EcoRIF (GAATTCCCTGCTGTACTAGCCACC) and HVISalR (with an N-terminal GFP cloned BamHI using primers GFPBamF and GFPBamR; Supplementary Fig. 1a). The myo6 heavy chain was co-expressed with human calmodulin and purified as described in detail in our previous work^18,39^. In brief, myo6 full-length and the Head construct were expressed in a 500-ml culture of SF21 cells (ThermoFisher Scientific B82101) and infected with a combination of baculovirus motor protein (multiplicity of infection (MOI) 3) and calmodulin (MOI 2) and grown at 27 °C for 72 h. Cells were pelleted and resuspended in phosphate-buffered saline (PBS) plus protease inhibitors (Roche cOmplete EDTA free). This was then sonicated for 3 min and spun at 70,000 × *g* (RCF) for 30 min at 4 °C. The supernatant was immediately loaded onto a 5-ml HisFF column (GE Healthcare). An AKTA programme was used with PBS and His high (50 mM Tris-HCl pH 7.5, 400 mM Imidazole, 300 mM NaCl) using 2 steps, the 5% step removed non-specific proteins and a 50% step eluted the myo6. The peak fraction collections were pooled, 20% glycerol added and snap frozen and stored at −80 °C. The Tail construct was expressed in BL21 one shot (DE3) cells (Invitrogen C600003). Two litres of cells grown in 2 × TY media at 37 °C to an OD600 of 0.6 were induced with 1 mM IPTG and grown overnight at 24 °C. Cells were pelleted at 8000 × *g* (RCF). The cell pellet was resuspended in His low (50 mM Tris-HCl (pH 7.5), 40 mM Imidazole, 300 mM NaCl) and sonicated for 5 min. This was then clarified at 70,000 × *g* (RCF) for 30 min and the supernatant was loaded onto the AKTA. Using His low and His high, a 5, 10 and 50% step programme was used to elute the protein. The 50% fraction peak was snap frozen and stored at −80 °C.

### Lipid bilayer on mica

A few monolayer thick, freshly cleaved mica sheet was deposited onto a glass coverslip using an index matching immersion oil and the coverslip made into a 300-μl flow cell. A supported lipid bilayer was formed using single unilamellar vesicle fusion as described^40^. The vesicles were made from DOPC (Sigma Aldrich™) and 1,2-dioleoyl-sn-glycero-phosphoethanolamine-*N*-Cyanine 5 (DOPE-Cy5, Avanti Polar Lipids™) at a 4000:1 ratio. In short, the vesicles (~1.7 mg ml^−1^ DOPC) in lipid buffer (LB; 20 mM Hepes pH 7.5 and 150 mM NaCl) were applied to the mica surface and allowed to fuse for 10 min at room temperature (RT) by adjusting the buffer to contain (in mM): 3.3 CaCl_2_, 100 Tris (pH 7.5), and 50 NaCl. Unfused vesicles were removed from the bilayer by washing with assay buffer (AB) containing (in mM): 25 Imidazole pH 7.4, 25 KCl, 4 MgCl_2_, and 1 EGTA. The formation of a continuous and fluid bilayer on the mica surface was confirmed by FRAP.

### Fluorescence microscopy in TIRF mode

The flowers were recorded using a Nikon Ti-Eclipse combining TIRFM including an EMCCD (Andor iXon3) camera and an A1 confocal module with a ×100 oil immersion objective, NA 1.49. A 10:1 ratio of myo6:GFP-myo6 was used and the 488-nm excitation laser set to 60 mW. To determine the density of myo6 along the perimeter of the flower (Fig. 1b), the perimeter of individual flowers, the total fluorescence intensity of individual flowers (*I*_total_) and the fluorescence signal of single GFP-myo6 molecules bound to the lipid bilayer (*I*_single GFP_) were measured in TIRF mode (Supplementary Fig. 1c, d). Here the flower perimeter was determined as the closed line connecting the pixels with maximum fluorescence intensity of a given flower (Supplementary Fig. 1c, yellow line). This closed line was enlarged radially by 400 nm in order to measure *I*_total_ of the individual flower (Supplementary Fig. 1c, area within green line). To reduce the effect of bleaching, only the first image acquired after activation of the excitation laser was used. *I*_single GFP_ was determined from binding events followed by single-step photo-bleaching (Supplementary Fig. 1d, fluorescence signal 18,917 ± 5523 (mean ± s.d., *n* = 288). The maximum *I*_total_ measured for each flower was within the linear range of the camera. Using *I*_total_ of individual flowers, *I*_single GFP_, the flower perimeter, and taking the 1:10 labelling ratio of GFP-myo6:myo6 into account, we estimated the average distance 3.74 ± 0.64 nm (mean ± s.d., *n* = 48) between neighbouring myo6 molecules along the flower perimeter.

### Fluorescence recovery after photobleaching

To test the fluidity of the bilayer, a circular area of ~40 μm in diameter was bleached in confocal mode using a 647-nm laser at 30 mW and FRAP was monitored using the same laser at 4 mW and an image sampling rate of 12 images per minute. All bilayers used in this study were tested using FRAP to ensure a full fluorescence recovery (FR) within <5 min. The diffusion coefficient *D* for DOPC in the absence and presence of myosin was estimated from the FR of a small area of ~4 μm in diameter in the centre of the bleached area. Single exponential fitting of the FR in the absence of myosin yielded a time constant *τ* consistent with *D* ~ 1.15−1.8 μm^2^ s^−1^ and in agreement with the literature for a supported and fluid DOPC bilayer^23,41^.

### Motility assay

A fluid DOPC bilayer was generated as described above. The formation of flowers was induced by incubation with 50 nM unlabelled myo6 and the flowers allowed to grow for 15 min. The buffer in the flow cell was replaced by AB buffer including 50 μg ml^−1^ bovine serum albumin (BSA, Sigma) and incubated for 5 min to prevent F-actin from binding to mica exposed at the membrane pores. Finally, the solution was replaced by AB buffer including 2 mM ATP, an oxygen scavenger system (50 mM dithiothreitol (DTT) and (in mg ml^−1^) 0.25 glucose oxidase, 7.5 glucose, 0.05 catalase) and ~17 nM Alexa488-phalloidin labelled F-actin (labelled at a 1:1 molar ratio). The motility of F-actin was recorded and analysed using TIRF microscopy as described in detail in our previous work^18,42^. In brief, time-lapse microscopy was performed and the movement of the actin filaments recorded at a frame rate of 0.1 Hz (for 600 s) using a ×100 TIRF microscope objective. The gliding velocity of F-actin was calculated using the analysis software GMimPro (www.mashanov.uk). The motility assays were all performed at 2 mM ATP and at 22 °C.

### Giant unilamellar vesicles

The GUVs are produced using electroformation as described in detail in our previous work^43^. In brief, 2 mg ml^−1^ DOPC, 2 μg ml^−1^ DOPE-Cy5 and 2 μg ml^−1^ Biotin-DHPE, dissolved in chloroform, were applied to a platinum wire and vesicle formation was induced in AB buffer supplemented with 100 mM sucrose to reach a total osmolarity of 200 mOsmol. The detaching vesicles were harvested and transferred to a streptavidin-coated flow cell using AB-buffer supplemented with 150 mM glucose to reach 250 mOsmol. Finally, the buffer was replaced by either 50 nM GFP-myo6 or 50 nM GFP alone in AB buffer including 150 mM glucose, and the change in fluorescence inside the tethered vesicle was monitored using confocal microscopy (Supplementary Fig. 1e). The time constant *τ* = 78 ± 19 s (mean ± s.d., *R*^2^ = 0.94 ± 0.04; *n* = 5), derived from the time course of the fluorescence increase inside the vesicles (11.24 ± 3.35 μm diameter, mean ± s.d., *n* = 5), and the application of Fick’s law allowed us to estimate the combined radius of the pores (~10 ± 5 nm, mean ± s.d.) introduced into the GUV vesicle membrane by membrane binding of GFP-myo6.

### Super resolution microscopy

The fluorescence images recorded on a Nikon Ti-Eclipse in TIRF mode were analysed using a combination of the proprietary N-STORM plugin and ImageJ. Our approach is related to the STORM/PALM techniques, e.g. ref. ^44^ in the sense that wide-field microscopy is used and the position of individual fluorophores is estimated by a two-dimensional (2D) Gaussian fit to the detected fluorescence signal. In contrast to these techniques, however, which involve individual fluorophores to be activated by a laser pulse, the fluorophores in our study are all activated, but only those GFP-myo6 molecules that are binding to the membrane are detected. This approach is related to the PAINT technique^45^. In brief, the algorithm automatically detected the centre position of local fluorescence intensity maxima by fitting a 2D Gaussian. Single GFP-myo6 molecules could be localised with a resolution of ~25 nm (full width at half maximum of the fitted Gaussian), at distances below the diffraction limit of ~200 nm. The resulting image consisted of a set of discs each centred according to the Gaussian fits. The SRM image consisted of the position of all fluorophores detected within given time intervals. The time intervals were encoded by different colours (Fig.2). A 10:1 ratio of myo6:GFP-myo6 was used for all myo6 concentrations we applied. Images were recorded at a frame rate of 1.6 images per second using an Andor iXon 3, EM gain 300x.

### Perimeter and area of the flowers measured using SRM

To determine the perimeter and area of individual flowers at time point *t*_*x*_ in SRM, we developed an algorithm based on the Nikon N-STORM plugin to analyse the SRM images. The detected fluorophores were fitted by a 2D Gaussian (15–20 nm radius). SRM images of ‘filled-in’ flowers were generated by overlaying all GFP-myo6-binding events detected from the beginning of the data acquisition until the time point *t*_*x*_. The ‘filled-in’ flowers formed an enclosed area of Gaussian spots (Supplementary Fig. 1f). The outline of this area was used to determine the perimeter of the flower at time point *t*_*x*_. Gaussian spots not connected to the enclosed area were ignored. The perimeter of the flowers in SRM was determined by connecting the centre positions of the Gaussian spots at the outline of the enclosed area. The procedure corresponds to low pass filtering (Gaussian spots 15–20 nm radius) and thresholding (eliminating Gaussian spots not connected to the enclosed flower area). To compensate for the delayed start of some flowers relative to others, each temporal evolution of a given flower was timeshifted to achieve synchronisation. The statistics are plotted for different concentrations of GFP-myo6 (50, 150, 300 nM; 19, 42, 55 flowers analysed, respectively). Owing to the higher numbers of flowers forming per field of view at higher myo6 concentrations in solution, the confluence of flowers became the limiting factor when measuring the perimeter of the flowers over time (Fig. 2c, 300 nM myo6).

### Gold nano-triangles

Gold nanoparticles were produced in a two-step reaction as described in detail in our previous work^30,46^. In brief, the reaction of HAuCl_4_ mixed with Na_2_S was followed spectroscopically to obtain triangles (equilateral prisms) with ~60–80 nm side length and ~8 nm thickness and stopped by the addition of an excess of Na_2_S. Following precipitation in 0.6 M cetyl trimethyl ammonium chloride and purification using 3 centrifugation cycles, the nano-triangle stock solution was centrifuged and the pellet resuspended in destilled water. Unless stated otherwise, the particles were applied to the mica surface at ~0.3 pM concentration, which led to ~50 particles on a 80 × 80 μm^2^ surface. When applied to the mica surface together with the DOPC bilayer in our experiments, the smallest distance of ~100 nm between the earliest myo6 hotspots (Fig. 3e) indicated that initially the proximity between bilayer and nano-triangle (60–80 nm side length) must have been ≤10–20 nm.

### In situ labelling of the gold nano-triangles using TRITC

TRITC-maleimide (Sigma) was dissolved in dimethylsulfoxide (Sigma) to a concentration of 470 μM. The solution was reacted with 10 mM DTT (Sigma) for 10 min at RT and diluted in LB to obtain a 100-μM thiolated dye solution. Gold nano-triangles diluted to 0.07–1.2 pM in LB buffer were added to a flow cell and left to sediment on the mica surface for 5 min before DOPC vesicles were added to form a bilayer as described above. After 10 min, the bilayer was washed three times with AB buffer supplemented with scavenger. The solution was exchanged by 100 μM thiolated dye in AB buffer and incubated for 10 min. Finally, the flow cell was washed ten times to ensure that all unbound dye was removed.

### Gold nano-particle effect on bilayer formation

The nano-triangles deposited on the mica surface in the absence of (white bars) or before a lipid bilayer was formed (grey bars) (Supplementary Fig. 3a). The number of detected triangles per field of view scaled roughly with the concentration of triangles applied. No significant difference was found between the number of triangles detected in the absence and presence of a lipid bilayer, indicating that the triangles were not covered but surrounded by the lipids. The relatively broad fluorescence intensity distribution from the labelled triangles was consistent with multiple dye molecules binding to a single triangle. Photobleaching of the spots was rarely observed in a single step. To probe the proximity of the lipid bilayer to the triangles, a line scan across an area with three detected triangles in the TRITC-channel (blue curve) was compared with the signal of the lipid Cy5-channel (red curve) (Supplementary Fig. 3b). No signal change within the resolution limit was found at the position of the nano-triangles, consistent with a proximity between lipid and triangles of ≤160 nm (one pixel). FRAP studies confirmed the fluidity of the bilayer in the presence of 0.3 pM nano-triangles, with *D* ~ 1.29 ± 0.1 μm^2^ s^−1^ (mean ± s.d., *n* = 2, *R*^2^ = 0.97 ± 0.005; Supplementary Fig. 3c). The nano-triangles acted as seeds for the formation of myo6-induced flowers. The number of flowers increased with the number of triangles applied and reached a ten-fold increase at 0.3 pM nano-triangles (Supplementary Fig. 3d).

### Geometric curve evolution

As discussed earlier, the time evolution of the planar curve *Γ*(*t*) is solely determined by the normal velocity (Eq. (1)). This mesoscale approach is used in many other disciplines, including geometry^47–49^, crystal growth^35–38,50,51^, combustion^52–54^ and fluid dynamics^37,55,56^. The basic idea is to model the dynamics through a semi-phenomenological growth law, i.e. by the dependence of the normal velocity on the position along the curve and the conformation of the curve. Before specifying this growth law, we need to introduce some basic concepts from the differential geometry of planar curves^57^. The local conformation of a planar curve *Γ* is determined by the curvature *κ* (*σ*):

4 $$\begin{array}{*{20}{c}} {{\hat{\mathbf{n}}}\kappa \left( \sigma \right) = \frac{1}{{\sqrt {g\left( \sigma \right)} }}\partial _\sigma \left( {\frac{{{\vec{\mathbf{\tau }}}\left( \sigma \right)}}{{\sqrt {g\left( \sigma \right)} }}} \right)} \end{array}$$

Here ${\vec{\mathbf{\tau }}} = \partial _\sigma {\vec{\mathbf{x}}}$ denotes the tangent vector to the curve *Γ*, and the metric is given by $g\left( \sigma \right) = \partial _\sigma {\vec{\mathbf{x}}} \cdot \partial _\sigma {\vec{\mathbf{x}}} = \left( {\partial _\sigma x} \right)^2 + \left( {\partial _\sigma y} \right)^2$. The unit tangent vector is then given by ${\hat{\mathbf{\tau }}} = {\vec{\mathbf{\tau }}}/\sqrt {g(\sigma )}$ . We use the convention that *κ* < 0 for convex portions of *Γ*, i.e. outward bulges along the front (Supplementary Fig. 4). The arc length *s* is defined as the length along the curve:

5 $$\begin{array}{*{20}{c}} {s\left( {t,\sigma } \right) = \mathop {\smallint }\nolimits_0^\sigma \sqrt {g\left( {\sigma \prime } \right)} {\mathrm{d}}\sigma \prime = \mathop {\smallint }\nolimits_0^\sigma \sqrt {\left( {\partial _\sigma x} \right)^2 \, +\, \left( {\partial _\sigma y} \right)^2} {\mathrm{d}}\sigma \prime } \end{array}$$

In the following, we use a parametrisation of the curve in terms of arc length. We assume that the membrane is spatially uniform in its properties and hence the normal growth velocity does not explicitly depend on the position ${\vec{\mathbf{x}}}$ of the curve but only on the curvature, *κ*, and gradients thereof, $\partial _s\kappa$. Moreover, we assume that there is no chirality, i.e. the system is invariant under the transformation *s* →−*s*. Then the growth velocity must be of the form:

6 $$\begin{array}{*{20}{c}} {V_{\mathrm{n}} = V_{\mathrm{n}}\left( {\kappa ,\partial _s^2\kappa , \ldots } \right)} \end{array}$$

We perform a gradient expansion keeping terms up to third order (note that *κ* has the dimension of an inverse length):

$$V_{\mathrm{n}} = {\it{\epsilon }}_0 - {\it{\epsilon }}_1\left( {\kappa - \kappa _0} \right) + {\it{\epsilon }}_2\left( {\kappa - \kappa _0} \right)^2 \, +\, {\it{\epsilon }}_3\left( {\kappa \, -\, \kappa _0} \right)^3 - \alpha \partial _s^2\kappa$$

7 $$\begin{array}{*{20}{c}} { \equiv f\left( \kappa \right) - \alpha \partial _s^2\kappa ,} \end{array}$$

where *∈*_*i*_, *κ*_0_ and *α* are phenomenological parameters with a sign convention that will become clear later as we discuss the physical significance of each term; we chose to expand with respect to *κ*_0_ for convenience as it allows us to shift the growth curve without changing its overall shape. Note that a term proportional to (∂_*s*_*κ*)^2^ is subleading compared to the terms contained in Eq. (7).

The first term, *∈*_0_ > 0, describes the basal curvature-independent growth speed due to the average rate of attachment of myo6 and the resulting displacement of lipid molecules. From the experimental data, we know that myo6 strongly favours saddle-shaped membrane geometry, i.e. negative curvature (*κ* < 0); see Fig. 3d, e. This tendency is reflected in the combined effect of the curvature-dependent terms (second to third term) that lead to a growth speed asymmetry favouring growth of convex interface regions with negative curvature. Without the third-order term, *∈*_3_(*κ* − *κ*_0_)^3^, the growth law, *V*_n_, would have a parabolic shape implying that the growth velocity diverges as *κ*→ -∞. This would lead to unphysical instabilities that create needle-like protrusions. The third-order term corrects for this and gives the N-shaped growth law shown in Fig. 4e where strong negative curvatures are attenuated. Taken together, the phenomenological parameters *∈*_1_, *∈*_2_ and *∈*_3_ characterise myo6 recruitment to saddle-shaped regions giving rise to the phenomenological growth law (Fig. 4e).

Finally, the last term, $- \alpha \partial _s^2\kappa$, penalises changes in the interface curvature, i.e. acts as a surface tension that smoothes the interface.

The physical motivation of this term was, to the best of our knowledge, first discussed in ref. ^58^ where the author studied the development of surface grooves at grain boundaries of polycrystalline materials. In these studies, it is assumed that newly deposited atoms from solution bind preferentially to valleys in the surface profile and the term $\alpha \partial _s^2\kappa$ is hence, on a microscopic level, interpreted as surface diffusion (Supplementary Fig. 5a). Similarly, in our system, smoothing of the interface is mediated by myo6 rearrangement along the protein–lipid interface and line tension of the lipid bilayer (Supplementary Fig. 5b). In ref. ^37^, the authors demonstrate that a term $\alpha \partial _s^2\kappa$ in phenomenological models of crystal growth acts as a short wavelength cut-off, analogous to surface tension in solidification processes. Furthermore, they show by a linear stability analysis that the dispersion relation of the growth rate of perturbations for crystal growth, pattern formation in multiphase fluid flow and phenomenological models equivalent to Eq. (7) share the same form: A morphological instability is induced by a driving force, which is counteracted by surface/line tension. In this analogy, one could interpret myo6 attachment and recruitment to saddle-shaped regions as the (chemical) driving force (the terms proportional to *∈*_0_, *∈*_1_, *∈*_2_ and *∈*_3_ in Eq. (7)), which is counteracted by the effective line tension term $\alpha \partial _s^2\kappa$.

### Parameters to describe the curvature-dependent growth rate

In order to determine the phenomenological parameters (∈_0_, *∈*_1_, *∈*_2_, *∈*_3_ and *κ*_0_) in the curvature-dependent growth rate, *f*(*κ*), we used the following approach: We measured the average radius, *R*_exp_(*t*), of a growing flower as a function of time and compared it to the theoretical results obtained for a growing spherical interface in the absence of shape fluctuations, i.e. we set $\partial _s^2\kappa = 0$ and *κ* = −1/*R* in Eq. (7) and solved the ordinary differential equation for the radius *R*:

8 $$\begin{array}{*{20}{c}} {\partial _tR = {\it{\epsilon }}_0 + {\it{\epsilon }}_1\left( {\frac{1}{R} + \kappa _0} \right) + {\it{\epsilon }}_2\left( {\frac{1}{R} + \kappa _0} \right)^2 \, -\, {\it{\epsilon }}_3\left( {\frac{1}{R} + \kappa _0} \right)^3} \end{array}$$

The initial condition, which we obtained from experimental data, was always set to *R*(0) = 0.3 μm. Supplementary Fig. 6 shows the average radius *R*_exp_ obtained from an ensemble of *n* = 253 growing flowers for a bulk concentration of *c* = 50 nM for myo6. The best fit of the data was obtained for the following parameter set: ${\it{\epsilon }}_0 = \left( {0.0171 \pm 0.0005} \right)\,\upmu {\mathrm{m}}\,{\mathrm{min}}^{ - 1}$, ${\it{\epsilon }}_1 = \left( {0.0102 \pm 0.0012} \right)\,\upmu {\mathrm{m}}^2\,{\mathrm{min}}^{ - 1}$, ${\it{\epsilon }}_2 = \left( {0.0010 \pm 0.0001} \right)\,\upmu{\mathrm{m}}^3\,{\mathrm{min}}^{ - 1}$, ${\it{\epsilon }}_3 = \left( {0.000403 \pm 0.000051} \right)\, \upmu {\mathrm{m}}^4\,{\mathrm{min}}^{ - 1}$, and $\kappa _0 = \left( {0.52 \pm 0.05} \right)\, \upmu {\mathrm{m}}^{ - 1}$.

### Parameter to describe the stiffness

The stiffness parameter *α*, as introduced in Eq. (7), is used as a fitting parameter such that the computational results for the wavelength in the flower pattern (see Fig. 3c) matches with the experimental results. We obtain

9 $$\begin{array}{*{20}{c}} {\alpha = \left( {1.00 \pm 0.25} \right) \times 10^{ - 6}\, \upmu {\mathrm{m}}^4{\mathrm{s}}^{ - 1}} \end{array}$$

### Numerical implementation of the model

To numerically solve for the time evolution of the closed planar curve, as described by Eq. (1), we chose the following algorithm following refs. ^36^^,37,51,55,56,59^. We discretise the curve *Γ* by a set of points (marker particles) and evaluate the velocity at each point. In order to prevent numerical instability^52,53^, it is important to choose a parametrisation in terms of arc length *s*, as it guarantees that points along the curve remain evenly distributed as the curve expands and deforms.

In detail, we adopt the following approach to solve the equations numerically. First, we decompose the local velocity into its component tangential and normal to the curve,

10 $$\begin{array}{*{20}{c}} {\partial _t{\vec{\mathbf{x}}} = V_{\mathrm{n}}{\hat{\mathbf{n}}} + T{\hat{\mathbf{\tau }}},} \end{array}$$

where $T = \partial _t{\vec{\mathbf{x}}} \cdot {\hat{\mathbf{\tau }}}$ and $V_{\mathrm{n}} = \partial _t{\vec{\mathbf{x}}} \cdot {\hat{\mathbf{n}}}$ are the tangential and normal components, respectively, with ${\hat{\mathbf{\tau }}} = \partial _s{\vec{\mathbf{x}}}$ the unit tangent vector and ${\hat{\mathbf{n}}}$ the unit normal vector to the curve *Γ*. As the time evolution and hence the shape of the interface is determined solely by the normal velocity *V*_n_ (cf. Eq. (1)), only the normal component of the velocity $\partial _t{\vec{\mathbf{x}}}$ is fixed. Hence, we can freely choose the velocity tangential to *Γ* without affecting the shape of the curve. For reasons that will become clear below, we require the following property to hold:

11 $$\begin{array}{*{20}{c}} {\partial _t{\vec{\mathbf{x}}}\left( {t,s} \right) \cdot \partial _s{\vec{\mathbf{x}}}\left( {t,s} \right)|_{s = 0} = 0} \end{array}$$

Geometrically, this condition means that we demand that the point at *s* = 0 moves along the normal to the curve for all times.

Now consider the arc length $s\left( {t,\sigma } \right) = \mathop {\smallint }\nolimits_0^\sigma \sqrt g {\mathrm{d}}\sigma \prime$ in terms of an arbitrary curve parametrisation *σ*, where $g = \partial _\sigma {\vec{\mathbf{x}}} \cdot \partial _\sigma {\vec{\mathbf{x}}}$ is the metric. Recalling that ${\hat{\mathbf{\tau }}} = \partial _s{\vec{\mathbf{x}}}$, $\partial _s{\hat{\mathbf{\tau }}} = \kappa {\hat{\mathbf{n}}}$ and $\partial _s{\hat{\mathbf{n}}} = - \kappa {\hat{\mathbf{\tau }}}$, the time derivative of the arc length can be rewritten as

12 $$\partial _ts\left( {t,\sigma } \right) = \mathop {\smallint }\nolimits_{\!\! 0}^\sigma \left( {\partial _t\partial _\sigma {\vec{\mathbf{x}}}} \right) \cdot \frac{{\partial _\sigma {\vec{\mathbf{x}}}}}{{\sqrt g }}{\mathrm{d}}\sigma \prime = \mathop {\smallint }\nolimits_{\!\! 0}^\sigma \left( {\partial _\sigma \left( {\partial _t{\vec{\mathbf{x}}}} \right)} \right) \cdot {\hat{\mathbf{\tau }}}{\mathrm{d}}\sigma \prime \begin{array}{*{20}{c}} { = \mathop {\smallint }\nolimits_0^\sigma \left( { - V_{\mathrm{n}}\kappa + \partial _sT} \right){\mathrm{d}}s\prime } \end{array}$$

Hence, the time evolution of the total length *L* (perimeter) of the curve can be written in the form

13 $$\begin{array}{*{20}{c}} {\partial _tL\left( t \right) = \mathop {\smallint }\nolimits_0^L \left( { - V_{\mathrm{n}}\kappa + \partial _sT} \right){\mathrm{d}}s = - \mathop {\smallint }\nolimits_0^L V_{\mathrm{n}}\kappa {\mathrm{d}}s,} \end{array}$$

where for the last equality in Eq. (13) we used the periodicity of *T* along the curve *Γ*. We now choose a parametrisation of the curve relative to the full arc length, *ρ* = *s*/*L* that is time invariant: ∂_*t*_(*s*/*L*) = 0; as the curve expands (*L* changes), the internal distance (*ρ*) between the points along the curve remains the same. In other words, the points on *Γ*(*t*) will be evenly distributed as the interface grows. With Eqs. (12) and (13), this condition translates into

14 $$\begin{array}{*{20}{c}} {T\left( {t,s} \right) - T\left( {t,0} \right) = \mathop {\smallint }\nolimits_0^s V_{\mathrm{n}}\kappa {\mathrm{d}}s\prime - \frac{s}{L}\mathop {\smallint }\nolimits_0^L V_n\kappa {\mathrm{d}}s} \end{array}$$

Since the choice, Eq. (11), amounts to *T*(*t*, 0) = 0, we have

15 $$\begin{array}{*{20}{c}} {T\left( {t,s} \right) = \mathop {\smallint }\nolimits_0^s V_{\mathrm{n}}\kappa {\mathrm{d}}s\prime - \frac{s}{L}\mathop {\smallint }\nolimits_0^L V_{\mathrm{n}}\kappa {\mathrm{d}}s} \end{array}$$

The evolution equation for the Cartesian coordinates *x* and *y* then follow from Eqs. (10) and (15) as

16 $$\begin{array}{*{20}{c}} {\partial _tx\left( {t,s} \right) = V_{\mathrm{n}}\partial _sy + \partial _sx\left( {\mathop {\smallint }\nolimits_0^s V_{\mathrm{n}}\kappa {\mathrm{d}}s\prime - \frac{s}{L}\mathop {\smallint }\nolimits_0^L V_{\mathrm{n}}\kappa {\mathrm{d}}s} \right),} \end{array}$$

17 $$\partial _ty\left( {t,s} \right) = - V_{\mathrm{n}}\partial _sx + \partial _sy\left( {\mathop {\smallint }\nolimits_0^s V_{\mathrm{n}}\kappa {\mathrm{d}}s\prime - \frac{s}{L}\mathop {\smallint }\nolimits_0^L V_{\mathrm{n}}\kappa {\mathrm{d}}s} \right)$$

To proceed, we derive an equation of motion for the angle *θ* between the tangent to the curve and the positive *x* axis, defined as

18 $$\begin{array}{*{20}{c}} {\partial _sx = \cos \theta ,} \end{array}$$

19 $$\begin{array}{*{20}{c}} {\partial _sy = \sin \theta } \end{array}$$

To this end, we will need the following identity

20 $$\partial _t\partial _s = \partial _t\left( {\frac{1}{{\sqrt g }}\partial _\sigma } \right) = \frac{1}{{\sqrt g }}\partial _t\partial _\sigma - \frac{1}{2}\frac{{\partial _tg}}{{g^{\frac{3}{2}}}} = \partial _s\partial _t - \left( { - V_{\mathrm{n}}\kappa + \partial _sT} \right)\partial _s,\begin{array}{*{20}{c}} { = \partial _s\partial _t - \frac{{\partial _tL}}{L}\partial _s,} \end{array}$$

where in the first line we used that $\partial _tg = 2g\left( { - V_{\mathrm{n}}\kappa + \partial _sT} \right)$, which follows from Eq. (12) together with the definition $\partial _ts = \mathop {\smallint }\nolimits_0^\sigma \frac{1}{2}\frac{{\partial _tg}}{{\sqrt g }}{\mathrm{d}}\sigma \prime$. Using Eq. (15) for $\partial _sT$, we arrive at Eq. (20). Furthermore, note that by definition $\partial _t{\hat{\mathbf{\tau }}} = \partial _t\partial _s{\vec{\mathbf{x}}} = - \partial _t\theta \cdot {\hat{\mathbf{n}}}$. Applying Eq. (20) to $\partial _t\partial _s{\vec{\mathbf{x}}}$ and using the fact that the curvature *κ* can be written in the form $\kappa = \partial _s^2x\partial _sy - \partial _sx\partial _s^2y = - \partial _s\theta$, we find:

21 $$\begin{array}{*{20}{c}} {\partial _t\theta \left( {t,s} \right) = - \partial _sV_{\mathrm{n}} - \partial _s\theta \left( {\frac{s}{L}\mathop {\smallint }\nolimits_0^L V_{\mathrm{n}}\partial _s\theta {\mathrm{d}}s - \mathop {\smallint }\nolimits_0^s V_{\mathrm{n}}\partial _s\theta {\mathrm{d}}s\prime } \right)} \end{array}$$

The position vector ${\vec{\mathbf{x}}}$ can be obtained from the definition $\partial _s{\vec{\mathbf{x}}} = {\hat{\mathbf{\tau }}}$ by integration: ${\vec{\mathbf{x}}} = {\vec{\mathbf{x}}}\left( {t,0} \right) + \mathop {\smallint }\nolimits_0^s {\hat{\mathbf{\tau }}}\,ds\prime$. The time-dependent integration constant ${\vec{\mathbf{x}}}(t,0)$ follows from Eq. (11): $\partial _t{\vec{\mathbf{x}}}\left( {t,0} \right) = V_{\mathrm{n}}\left( {t,0} \right) \cdot {\hat{\mathbf{n}}}(t,0)$, and hence ${\vec{\mathbf{x}}}\left( {t,0} \right) = {\vec{\mathbf{x}}}\left( {0,0} \right) + \mathop {\smallint }\nolimits_0^t V_{\mathrm{n}}\left( {t\prime ,0} \right) \cdot {\hat{\mathbf{n}}}\left( {t\prime ,0} \right)dt\prime$. The solution of Eq. (21) can then be used to reconstruct the position vector:

22 $$\begin{array}{*{20}{c}} {x\left( {t,s} \right) = x\left( {0,0} \right) + \mathop {\smallint }\nolimits_0^t V_{\mathrm{n}}\left( {t\prime ,0} \right)\sin \theta \left( {t\prime ,0} \right){\mathrm{d}}t\prime + \mathop {\smallint }\nolimits_0^s \cos \theta \left( {t,s\prime } \right){\mathrm{d}}s\prime ,} \end{array}$$

23 $$\begin{array}{*{20}{c}} {y\left( {t,s} \right) = y\left( {0,0} \right) - \mathop {\smallint }\nolimits_0^t V_{\mathrm{n}}\left( {t\prime ,0} \right)\cos \theta \left( {t\prime ,0} \right){\mathrm{d}}t\prime + \mathop {\smallint }\nolimits_0^s \sin \theta \left( {t,s\prime } \right){\mathrm{d}}s\prime } \end{array}$$

Finally, using the rescaled curve parameter *ρ* = *s*/*L*, the set of equations read in summary:

24 $$\begin{array}{*{20}{c}} {\partial _t\theta \left( {t,\rho } \right) = - \frac{1}{L}\partial _\rho V_{\mathrm{n}} - \frac{1}{L}\partial _\rho \theta \left( {\rho \mathop {\smallint }\nolimits_0^1 V_{\mathrm{n}}\partial _\rho \theta {\mathrm{d}}\rho - \mathop {\smallint }\nolimits_0^\rho V_{\mathrm{n}}\partial _\rho \theta {\mathrm{d}}\rho \prime } \right),} \end{array}$$

25 $$\begin{array}{*{20}{c}} {\partial _tL\left( t \right) = \mathop {\smallint }\nolimits_0^1 V_{\mathrm{n}}\partial _\rho \theta {\mathrm{d}}\rho ,} \end{array}$$

26 $$\begin{array}{*{20}{c}} {x\left( {t,\rho } \right) = x\left( {0,0} \right) + \mathop {\smallint }\nolimits_0^t V_{\mathrm{n}}\left( {t\prime ,0} \right)\sin \theta \left( {t\prime ,0} \right){\mathrm{d}}t\prime + L\mathop {\smallint }\nolimits_0^\rho \cos \theta \left( {t,\rho \prime } \right){\mathrm{d}}\rho \prime ,} \end{array}$$

27 $$\begin{array}{*{20}{c}} {y\left( {t,\rho } \right) = y\left( {0,0} \right) - \mathop {\smallint }\nolimits_0^t V_{\mathrm{n}}\left( {t\prime ,0} \right)\cos \theta \left( {t\prime ,0} \right){\mathrm{d}}t\prime + L\mathop {\smallint }\nolimits_0^\rho \sin \theta \left( {t,\rho \prime } \right){\mathrm{d}}\rho \prime .} \end{array}$$

We solve the partial integro-differential equation Eq. (24) and the ordinary differential equation Eq. (25) using the Finite-Element software *COMSOL 5.3a*. The coupled system of equations is solved on a line $\rho \in \left[ {0,1} \right]$ with boundary and initial conditions as follows: For the *θ* equation, Eq. (24), we impose Dirichlet boundary condition at the end of the interval $\theta \left( {t,1} \right) = \theta \left( {t,0} \right) + 2{{\pi }}$ since the angle *θ* must rotate by 2π when traversing the closed curve.

As the initial condition for the curve, we take a circle that is perturbed by a finite number of Fourier modes

28 $$\theta \left( {0,\rho } \right) = 2\pi \rho + \delta \sum\limits_{n = 1}^N g \left( n \right)\sin \, \left ( 2{\mathrm{\pi }}n\rho + u\left( n \right) \right),$$

where *δ* = 0.03 is a parameter for the amplitude of perturbations. The functions *g*(*n*) and *u*(*n*) are random numbers drawn from a Gaussian distribution with mean zero and standard deviation of 1 and a uniform distribution on the interval [0, π], respectively. This way we generate circular seeds with some initial random surface roughness.

To simulate the time evolution of a triangular seed, we only need to select the corresponding mode, hence giving $\theta \left( {0,\rho } \right) = 2{{\pi }}\rho + \delta \sin \,\left ( {6{{\pi }}\rho } \right)$ where we set *δ* = 0.8 in this case (see Supplementary Fig. 7a, b for an illustration). The initial condition for the perimeter *L*(*t*) was set to *L*(0) = 2π*R*_0_, where *R*_0_ = 0.4 μm is the initial seed radius. The origin is located at (0, 0) and we therefore set *x*(0, 0) = 0 and *y*(0, 0) = −*R*_0_ (Supplementary Fig. 7b). Finally, we obtain the cartesian position, ${\vec{\mathbf{x}}}$, using Eqs. (26) and (27) from the solutions of *θ* and *L* in *COMSOL 5.3a*.

### Hotspot distance determined from the numerical simulation

The wavelength (average hotspot distance) 〈dz〉 of a growing flower (Fig. 3c) was obtained as follows: Given the numerical solutions of Eqs. (24)–(27), we calculated the average Euclidean distance between two neighbouring outward bulges (peak-to-peak distance) along the curve at a time (Supplementary Fig. 8a). In addition, we calculated the average flower radius *R* at each time step:

29 $$\begin{array}{*{20}{c}} {R\left( t \right) = \mathop {\smallint }\nolimits_0^1 \sqrt {x\left( {t,\rho \prime } \right)^2 \, + \, y\left( {t,\rho \prime } \right)^2} {\mathrm{d}}\rho \prime .} \end{array}$$

The result for ﻿〈dz〉 as a function of the average flower diameter is shown in Supplementary Fig. 8b (blue curve). As can be inferred from this Figure, the curve shows some irregularities but saturates (on average) at a value of ﻿〈dz〉 around 1 μm. The irregularities originate from two sources: (i) Tip splitting, which leads to an abrupt decrease of 〈dz〉 as one peak splits into two peaks, which have (initially) a smaller distance (see Supplementary Fig. 8a for an illustration). (ii) Averaging of the peak-to-peak distance along the curve, which produces minor irregularities between time steps.

To obtain a smooth curve and for better comparison with our experiments, we fitted the blue curve in Supplementary Fig. 8b using a fit function with four parameters of the form $p\left( x \right) = a\left( {1 - \exp \left( { - \frac{{x - b}}{c}} \right)} \right) + d$ (Supplementary Fig. 8b, green curve).

The theoretical curve for ﻿〈dz〉 appears to be shifted relative to the experimentally obtained hotspot distance (see Fig. 3c). The reason for this lies in the assumption of our model: The gradient expansion of the normal velocity Eq. (7) sets the smallest length scale, which can be resolved. More precisely, regions of too large negative curvature (or a too small initial seed) cannot grow but decay to a point as indicated from the shape of the growth function in Fig. 4e. In the experiments, however, we are able to measure distances between only two hotspots, which are roughly 100 nm apart (Fig. 3a, b). This stage cannot be captured by the model since the model is only valid around the onset of pattern formation, i.e. for large enough seeds that exhibit a wavelength. This leads to a slight shift of the theoretical curve for 〈dz〉.

We could, however, shift this length scale penalty in the model to smaller values by including higher-order terms in the gradient expansion Eq. (7), but this would also add more parameters to the model without gaining additional insight.

### Reporting summary

Further information on research design is available in the Nature Research Reporting Summary linked to this article.

## Supplementary information

Supplementary Information

Reporting Summary

Source data

## Data Availability

Data supporting the findings of this manuscript are available from the corresponding authors upon reasonable request. A reporting summary for this article is available as a Supplementary Information file. The source data underlying Figs. 1b, c, 2c and 3c and Supplementary Figs. 1e, 3a and 6 are provided as a Source Data file.
