# Supplementary material for: Reconstitution reveals how myosin-VI self-organises to generate a dynamic mechanism of membrane sculpting
Source: Nat Commun. 2019 Jul 24;10:3305. doi: 10.1038/s41467-019-11268-9 (PMC6656732; doi:10.1038/s41467-019-11268-9)
Supplement: Supplementary file 1 — Supplementary Information [file 41467_2019_11268_MOESM1_ESM.pdf]

Supplementary Information

Reconstitution reveals how myosin-VI self-organises to generate a dynamic mechanism of membrane sculpting

Rogez *et al.*

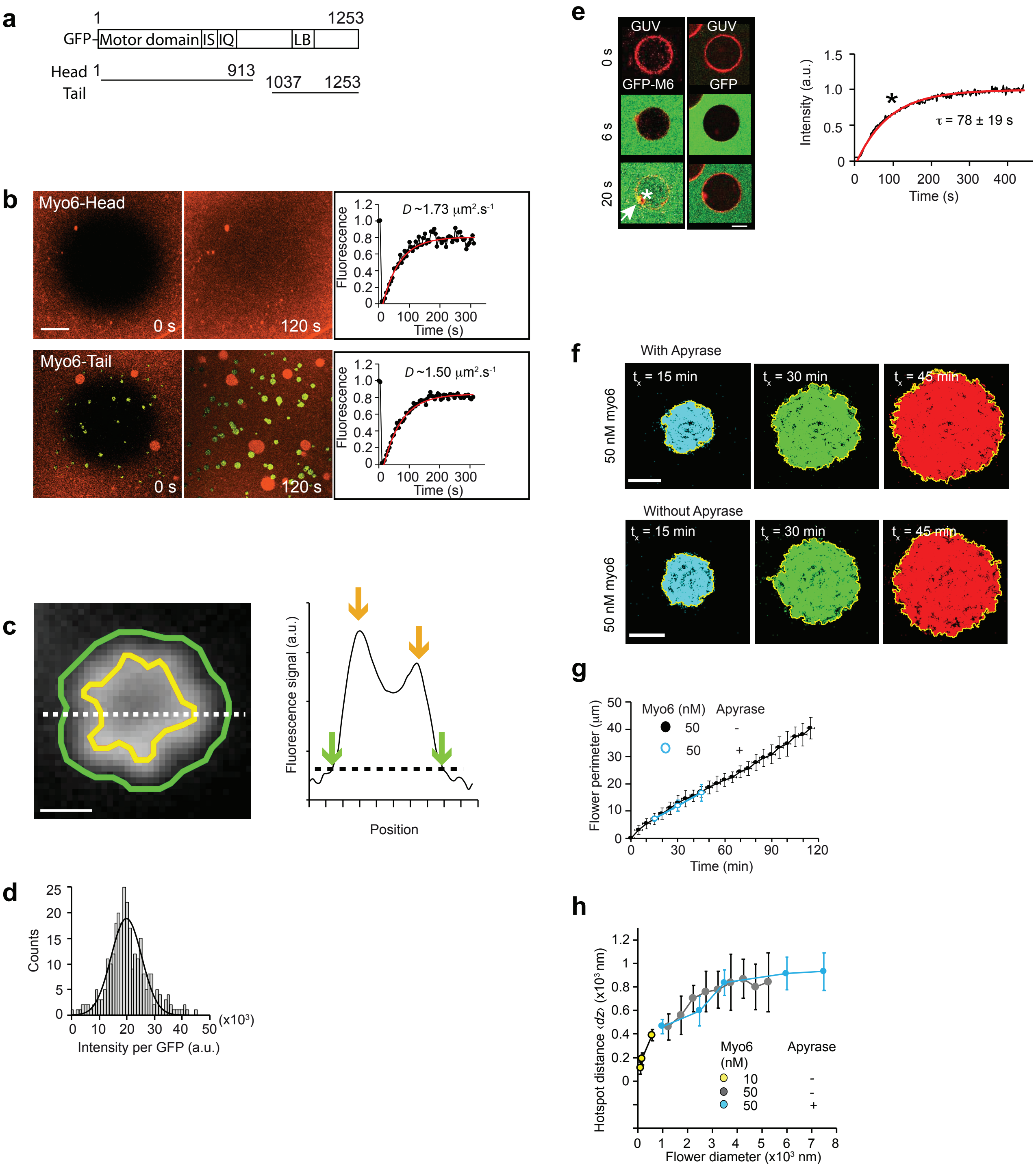

Supplementary Figure 1. The myo6 tail domain is responsible for membrane binding. **a**, Sequence and domain structure of human full-length myo6, myo6-head and tail constructs; IS (53-residue insert), IQ (calmodulin-binding motif) and LB (lipid-binding site aa1084-1099) (Ref (9)). **b**, Myo6-Head construct did not, while the Tail construct did bind to the DOPC bilayer (50 nM myo6 (Head/Tail), 10% GFP-myo6). FRAP experiments (DOPC:DOPE-Cy5, 4000:1) to determine the lipid diffusion coefficient D; with Myo6-Head (no binding) $\tau = 57.8$ s ($R^2 = 0.92$), $D \sim 1.73$ μm².s⁻¹; $D_{av} \sim 1.66 \pm 0.22$ μm².s⁻¹ (mean ± s.d., $n = 3$). With Myo6-Tail (binding) $\tau = 66.6$ s ($R^2 = 0.98$), $D \sim 1.50$ μm².s⁻¹; $D_{av} \sim 1.41 \pm 0.26$ μm².s⁻¹ (mean ± s.d., $n = 3$). **c**, TIRFM of a myo6 induced flower-shaped membrane pore (150 nM myo6,10% GFP-myo6). Pixels with local maximum fluorescence intensity were connected to form a closed line (yellow); total fluorescence intensity obtained from area within green line; scale bar 1 μm. **d**, Emission signal of single GFP-myo6; mean ± s.d. 18,917 ± 5,523 ($R^2 = 0.88$) for 288 GFP molecules photobleaching in a single step. **e**, Confocal microscopy of GUV vesicles (DOPC:DOPE-Cy5 (red), 4000:1) in the presence of 50 nM GFP-myo6 (GFP-M6, green) or 50 nM GFP (GFP, green; control experiment). The time constant τ for fluorescence increase inside the vesicles in the presence of 50 nM GFP-myo6, $\tau = 78 \pm 19$ s (mean ± s.d., $n = 5$, $R^2 = 0.94 \pm 0.04$; diameter of the vesicles 11.24 ± 3.35 μm). **f**, Determination of the perimeter of flowers in SRM; the signal is integrated fom the start of flower growth until time t_x; the detected fluorophores are plotted as Gaussian discs (20 nm radius). The perimeter is obtained by connecting the centre of the peripheral Gaussian spots of the enclosed area (yellow line). Representative examples of 50 nM myo6 (10% GFP-myo6) induced flower growth, with and without apyrase treatment of myo6 to remove residual nucleotide bound to the myo6 catalytic domain. **g**, The perimeter growth is not affected by myo6 apyrase treatment (data without apyrase from Fig. 2c; myo6 with apyrase treatment (blue unfilled circles) mean ± s.d., $n = 4$). **h**, The average hostpot distance $\langle dz \rangle$ is also not affected by myo6 apyrase treatment (data without apyrase from Fig. 3c; myo6 with apyrase treatment (blue filled circles) mean ± s.d., $n = 9$-19). Source data are provided as a Source Data file.

a

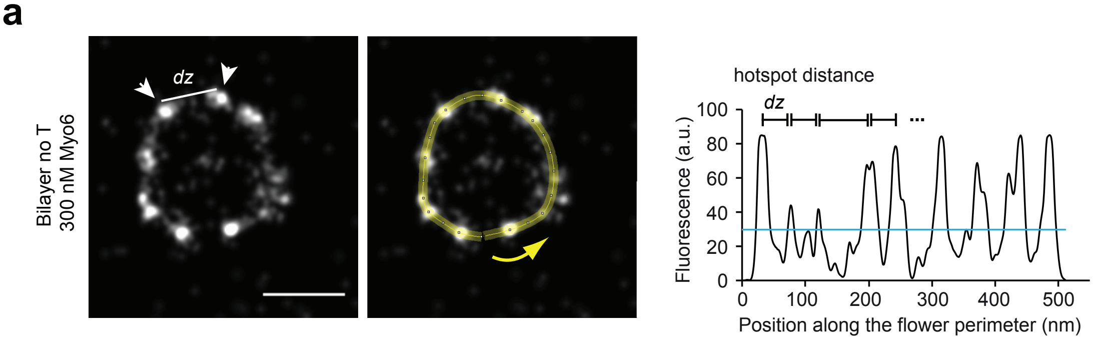

b

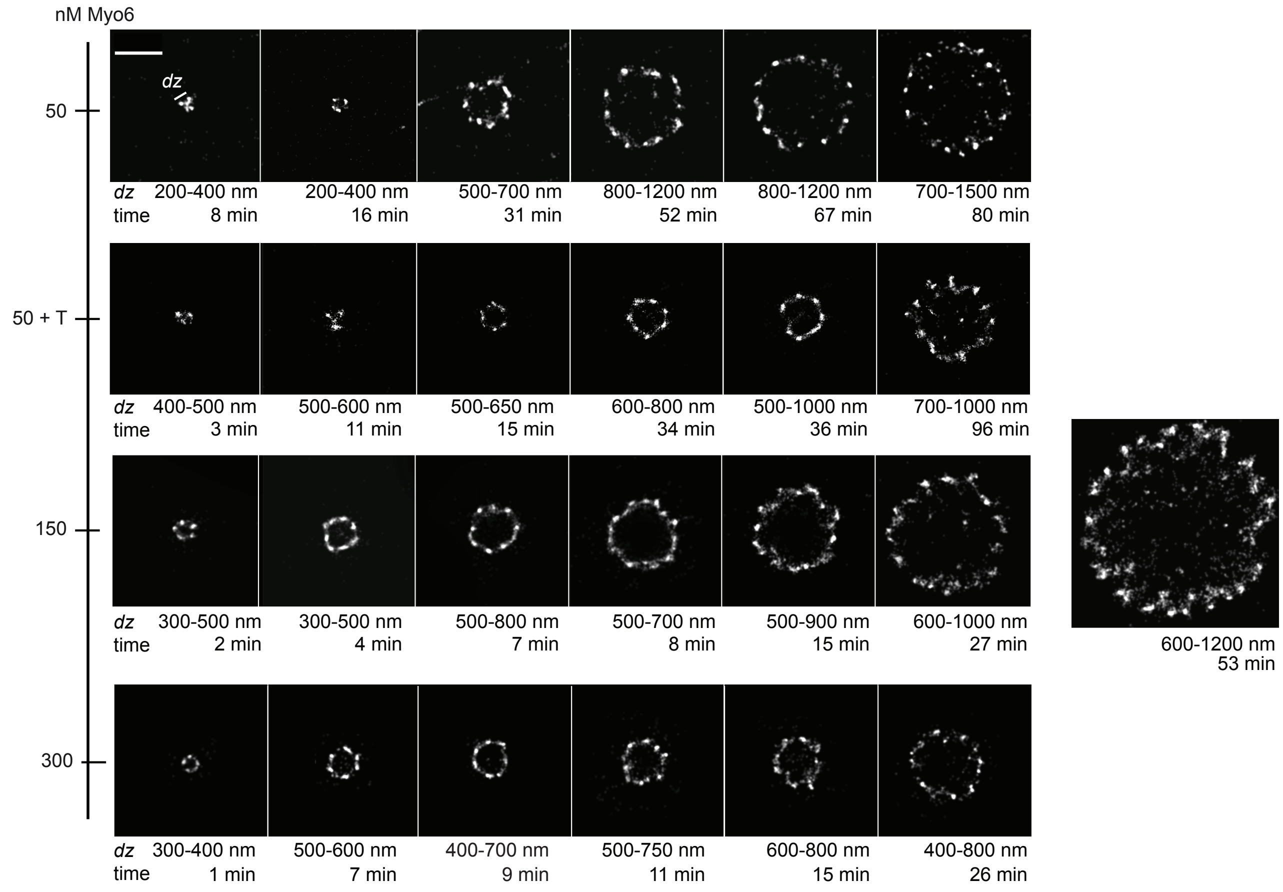

Supplementary Figure 2. Membrane pores formed at different myo6 concentrations. a, Analysis of the super resolution images; scale bar 10 μm. The distances *dz* between the hotspots were determined by thresholding the fluorescence intensity (blue line); the threshold was set at ~3 times the background fluorescence noise level. SRM integration time 1 min. **b**, Representative examples of super resolution images at different time points after addition of myo6; scale bar 2 μm; for the condition 50 + T we applied 0.3 pM triangles (T) to the surface before forming a lipid bilayer. SRM integration time 1 min, except for 300 nM myo6 (30s).

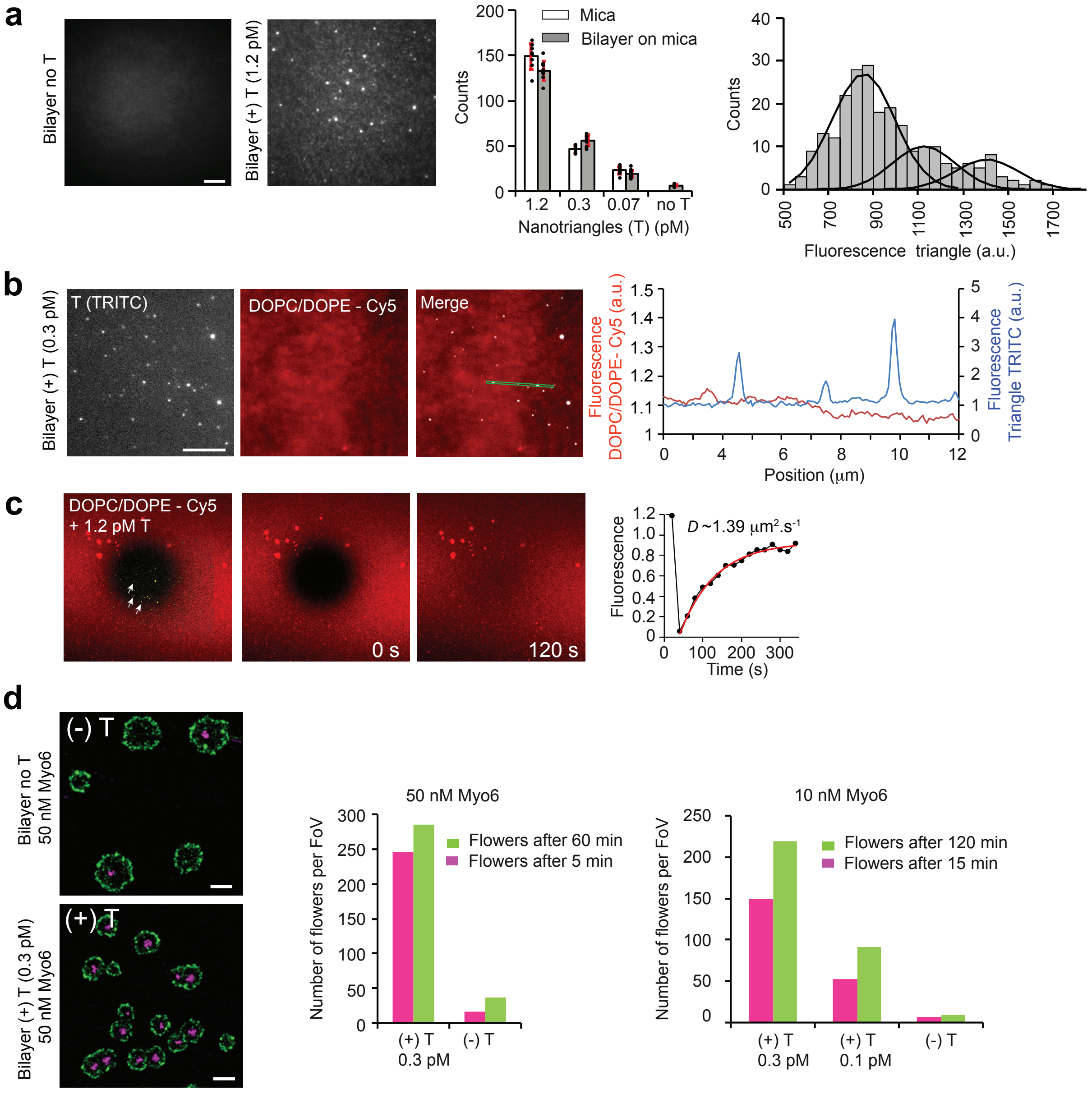

Supplementary Figure 3. Effect of nano-triangles on the bilayer and on myo6-induced flowers. a, TIRFM to detect gold nano-triangles deposited on a mica surface, labelled using TRITC-maleimide (Sigma); scale bar 10 μm. Nano-triangles labelled in the absence of (white bars), or after a lipid bilayer was formed (grey bars) and detected in the TRITC channel. Number of triangles detected (mean ± s.d. and raw data points) in 8 -14 fields of view (FoV) in each condition. No significant difference was found between *N* of detected triangles in the presence and absence of the bilayer (*p*-value 0.01, 0.002 and 0.02 for 1.2, 0.3 and 0.07 pM triangles respectively, two-tailed t-test). Fluorescence intensity (a.u.) of TRITC-labelled triangles; scale bar 10 μm. Intensity maxima at 825 ± 139, 1160 ± 140 and 1388 ± 122 (mean ± s.d.) consistent with one, two and three dye molecules respectively. Source data are provided as a Source Data file. **b**, Signal of a line scan (merged image, green box) across an area with 3 detected triangles in the TRITC-channel (blue curve) compared with the signal of the lipid Cy5-channel (red curve). **c**, FRAP studies (DOPC:DOPE-Cy5, 4000:1) to confirm the fluidity of the bilayer in the presence of the 1.2 pM nano-triangles (green, white arrows); for a bleached circle of 45 μm diameter $\tau = 95$ s ($R^2 = 0.98$), corresponding to $D \sim 1.39$ $\mu m^2.s^{-1}$. **d**, Number of flowers per FoV at different times after addition of myo6 with and without nano-triangles inserted into the bilayer; scale bars 2 μm.

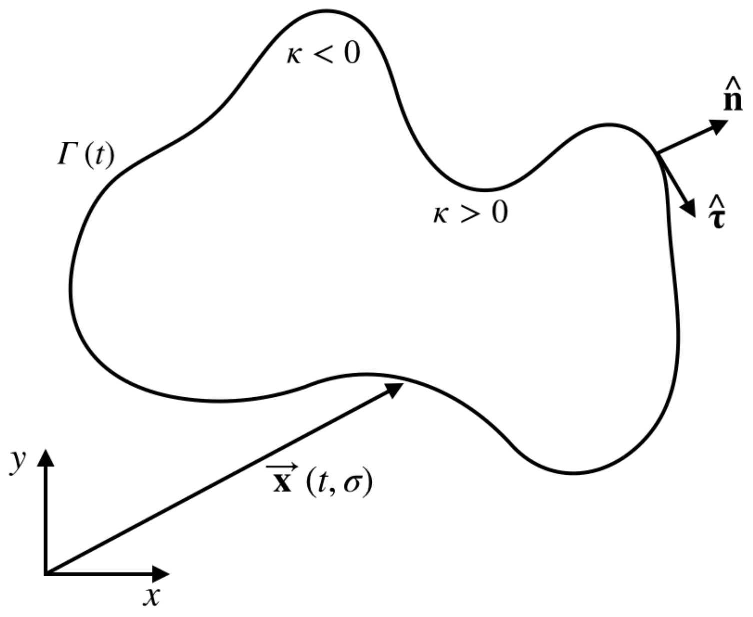

Supplementary Figure 4. Mathematical description of a closed planar curve. The curve $\Gamma(t)$ is parametrized by the Cartesian position vector $\vec{\mathbf{x}}(t,\sigma)$. The conformation of $\Gamma(t)$ is characterized by the curvature κ and we use the convention that κ is negative for convex portions of the curve.

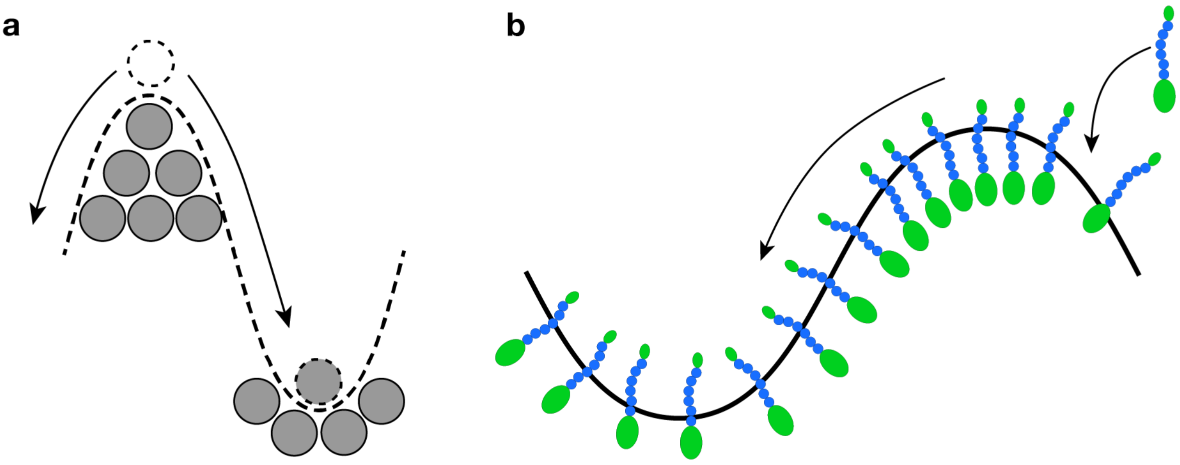

Supplementary Figure 5. Microscopic origin of the effective line tension term in the model. a, Surface diffusion of atoms (grey circle) along grain boundaries (dashed line) as introduced in Ref.[58]. Atoms from solution (dashed circle) bind and diffuse along the interface until they reach a valley where the free energy is minimized (dashed grey circle). This process leads naturally to smoothing of an irregular surface profile. **b**, Myosin6 binds to the protein-lipid interface (black solid line) and thereby increases the interface perimeter. The increase of the perimeter is counteracted by myosin6 rearrangement along the interface and line tension of the lipid. This also leads to smoothing of the surface profile analogous to surface growth models (**a**).

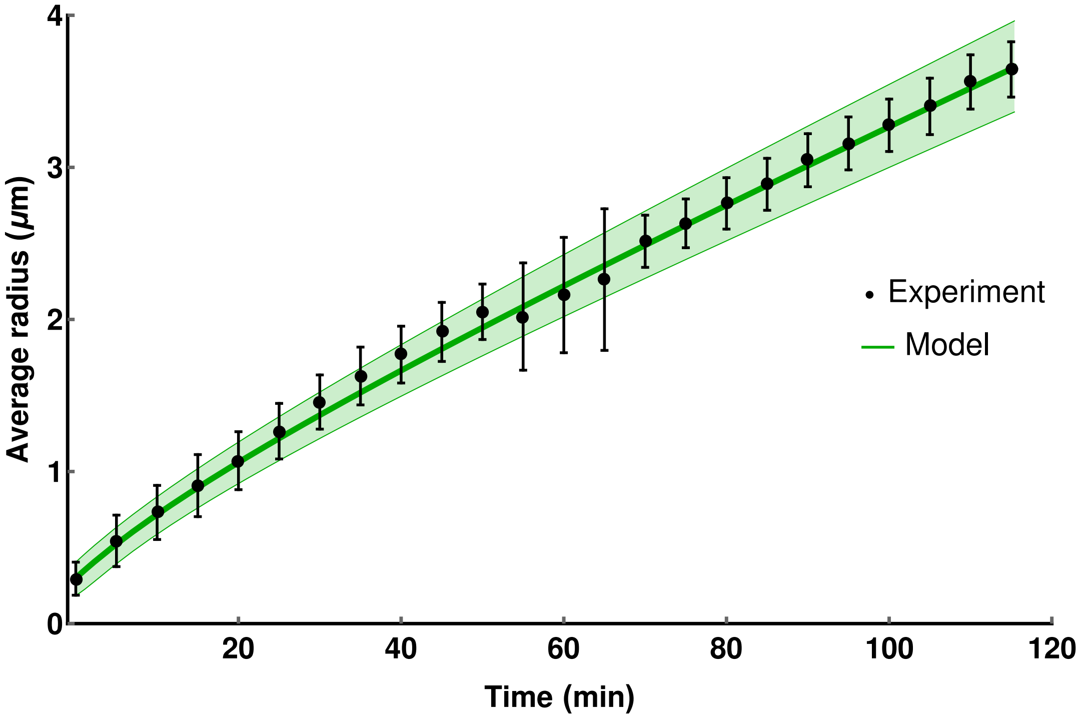

Supplementary Figure 6. Fit of the phenomenological parameters. Average radius $\langle R_{\mathrm{exp}} \rangle$ obtained for a bulk concentration $c = 50\ nM$ for myo6 from an ensemble of $N = 253$ measurements of the flower radius (black filled symbols). The (green) solid line shows the best theoretical result obtained from solving Eq.8. Source Data are provided as a Source Data file.

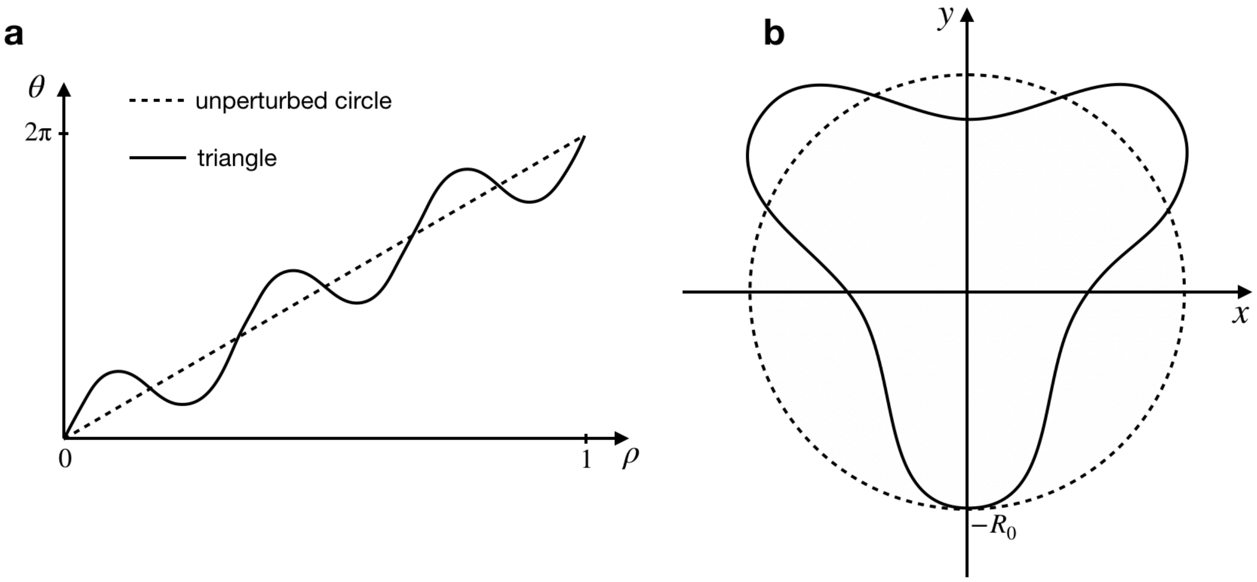

Supplementary Figure 7. Illustration of the initial configuration for the angle $\boldsymbol{\theta}$. **a**, Initial configuration of θ for an unperturbed circle (dotted line) and a triangle (solid line), respectively. **b**, The corresponding shapes in Cartesian coordinates for the initial conditions of θ as shown in panel **a**.

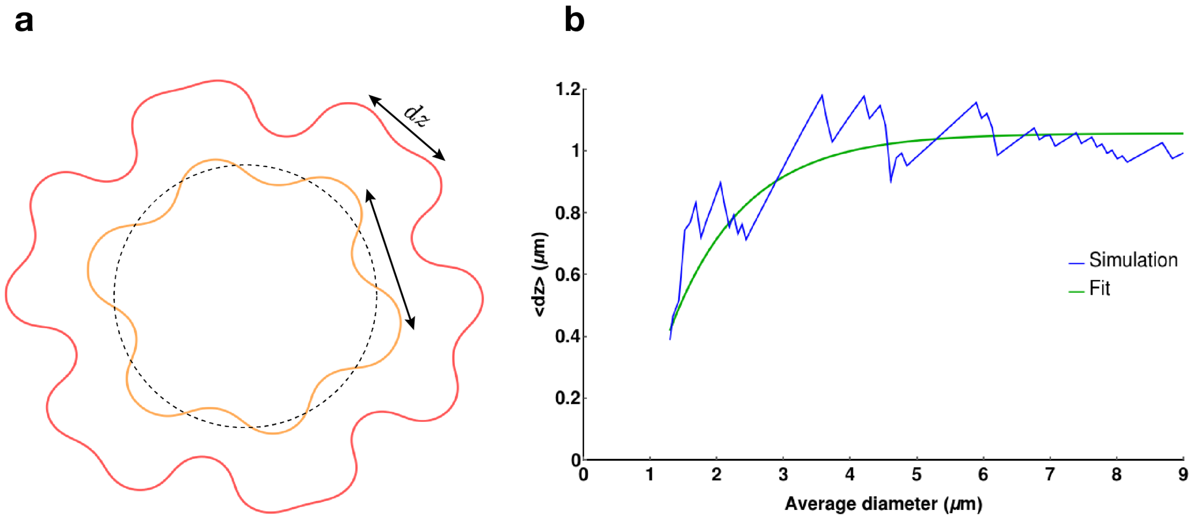

Supplementary Figure 8. Determination of the average hotspot distance. a, Shown is the simulated flower shape at two successive time points (orange and red curve, respectively). The hotspot distance $\langle dz \rangle$ is obtained from the average Euclidean distance between neighbouring outward bulges (black double arrow). The dotted circle represents the average diameter of the orange curve. **b**, Hotspot distance $\langle dz \rangle$ plotted as a function of the average flower diameter. The blue curve shows the result as obtained from the simulation data and the green graph is a smooth fit to this curve.
