# Supplementary material for: Reconstitution reveals how myosin-VI self-organises to generate a dynamic mechanism of membrane sculpting
Source: Nat Commun. 2019 Jul 24;10:3305. doi: 10.1038/s41467-019-11268-9 (PMC6656732; doi:10.1038/s41467-019-11268-9)
Supplement: Supplementary file 2 — Reporting Summary [file 41467_2019_11268_MOESM2_ESM.pdf]

nature research

Corresponding author(s): Erwin Frey and Claudia Veigel

Last updated by author(s): Jun 19, 2019

Reporting Summary

Nature Research wishes to improve the reproducibility of the work that we publish. This form provides structure for consistency and transparency in reporting. For further information on Nature Research policies, see Authors & Referees and the Editorial Policy Checklist.

Statistics

For all statistical analyses, confirm that the following items are present in the figure legend, table legend, main text, or Methods section.

n/a	Confirmed	
☐	☒	The exact sample size (*n*) for each experimental group/condition, given as a discrete number and unit of measurement
☒	☐	A statement on whether measurements were taken from distinct samples or whether the same sample was measured repeatedly
☐	☒	The statistical test(s) used AND whether they are one- or two-sided<br>*Only common tests should be described solely by name; describe more complex techniques in the Methods section.*
☒	☐	A description of all covariates tested
☒	☐	A description of any assumptions or corrections, such as tests of normality and adjustment for multiple comparisons
☐	☒	A full description of the statistical parameters including central tendency (e.g. means) or other basic estimates (e.g. regression coefficient) AND variation (e.g. standard deviation) or associated estimates of uncertainty (e.g. confidence intervals)
☐	☒	For null hypothesis testing, the test statistic (e.g. *F*, *t*, *r*) with confidence intervals, effect sizes, degrees of freedom and *P* value noted<br>*Give P values as exact values whenever suitable.*
☒	☐	For Bayesian analysis, information on the choice of priors and Markov chain Monte Carlo settings
☒	☐	For hierarchical and complex designs, identification of the appropriate level for tests and full reporting of outcomes
☐	☒	Estimates of effect sizes (e.g. Cohen's *d*, Pearson's *r*), indicating how they were calculated

Our web collection on statistics for biologists contains articles on many of the points above.

Software and code

Policy information about availability of computer code

Data collection	The fluorescence data were recorded on a commercially available Nikon Ti-Eclipse combining TIRF microscopy including an Andor iXon3 EMCCD camera and an A1 confocal module using proprietary software.
Data analysis	The fluorescence data were analysed using a combination of the proprietary N-STORM plug-in and ImageJ

For manuscripts utilizing custom algorithms or software that are central to the research but not yet described in published literature, software must be made available to editors/reviewers. We strongly encourage code deposition in a community repository (e.g. GitHub). See the Nature Research guidelines for submitting code & software for further information.

Data

Policy information about availability of data

All manuscripts must include a data availability statement. This statement should provide the following information, where applicable:

- Accession codes, unique identifiers, or web links for publicly available datasets
- A list of figures that have associated raw data
- A description of any restrictions on data availability

All data that support the findings of this study are contained within the paper and its Supplementary Information or are available from the corresponding authors upon reasonable request.

October 2018

Field-specific reporting

Please select the one below that is the best fit for your research. If you are not sure, read the appropriate sections before making your selection.

☒ Life sciences ☐ Behavioural & social sciences ☐ Ecological, evolutionary & environmental sciences

For a reference copy of the document with all sections, see nature.com/documents/nr-reporting-summary-flat.pdf

Life sciences study design

All studies must disclose on these points even when the disclosure is negative.

Sample size	The sample size was chosen such that 10% more data gave no significant change in the estimated value. Standard deviation values and histograms of the data are given in order to enable a direct classification of the quality of the data.
Data exclusions	No data were excluded from the analysis
Replication	The reproducibility of the data was ensured by repeating the experiments with at least three independent protein preparations with comparable results.
Randomization	All proteins used in this study are completely defined by their DNA sequence and are therefore not relevant for a randomisation process.
Blinding	The identity of all constructs and the process of analysis were clearly defined and therefore not relevant for a blinding process.

Reporting for specific materials, systems and methods

We require information from authors about some types of materials, experimental systems and methods used in many studies. Here, indicate whether each material, system or method listed is relevant to your study. If you are not sure if a list item applies to your research, read the appropriate section before selecting a response.

Materials & experimental systems

n/a	Involved in the study	
☒	☐	Antibodies
☐	☒	Eukaryotic cell lines
☒	☐	Palaeontology
☒	☐	Animals and other organisms
☒	☐	Human research participants
☒	☐	Clinical data

Methods

n/a	Involved in the study	
☒	☐	ChIP-seq
☒	☐	Flow cytometry
☒	☐	MRI-based neuroimaging

Eukaryotic cell lines

Policy information about cell lines

Cell line source(s)	Commercial Sf21 cells (Spodoptera frugiperda) from ThermoFisher (Cat No B82101) were used exclusively for the recombinant protein expression.
Authentication	A certificate of analysis provided by the manufacturer (cell growth and recovery; bacterial and fungal contamination)
Mycoplasma contamination	A certificate of analysis is provided by the manufacturer (mycoplasma contamination)
Commonly misidentified lines (See ICLAC register)	No commonly misidentified cell lines was used in this study.
